# Comparative Effectiveness of Integrated Peer Support and Clinical Staffing Models for Community-Based Residential Mental Health Rehabilitation: A Prospective Observational Study

**DOI:** 10.1007/s10597-022-01023-8

**Published:** 2022-09-03

**Authors:** Stephen Parker, U. Arnautovska, N. Korman, M. Harris, F. Dark

**Affiliations:** 1 School of Medicine, The University of Queensland, Brisbane, Australia; 2 Metro South Addiction and Mental Health Services, Woolloongabba, Australia; 3 Metro North Addiction and Mental Health Service, Chermside, Australia; 4 School of Public Health, The University of Queensland, Brisbane, Australia; 5 The Prince Charles Hospital, Chermside, QLD 4032 Australia

**Keywords:** Schizophrenia, Rehabilitation, Residential services, Peer support, Staffing model

## Abstract

This observational study compared the outcomes of consumers receiving community-based residential mental health rehabilitation support in Australia under a clinical staffing model and an integrated staffing model where Peer Support Workers are the majority component of the staffing profile. Reliable and clinically significant (RCS) change between admission and discharge in functional and clinical assessment measures were compared for consumers receiving care under the clinical (n = 52) and integrated (n = 93) staffing models. Covariate analyses examined the impact of known confounders on the outcomes of the staffing model groups. No statistically significant differences in RCS improvement were identified between the staffing models. However, logistic regression modelling showed that consumers admitted under the integrated staffing model were more likely to experience reliable improvement in general psychiatric symptoms and social functioning. The findings support the clinical and integrated staffing models achieving at least equivalent outcomes for community-based residential rehabilitation services consumers.

**Supplementary Information:**

The online version contains supplementary material available at 10.1007/s10597-022-01023-8.

## Introduction

Contemporary community-based mental health residential rehabilitation services combine medium-to-long term accommodation with intensive rehabilitation and psychosocial support (Parker et al., 2019a, 2019b, 2019c). These services provide transitional residential rehabilitation (TRR) to people experiencing severe and persistent mental illness to enable them to live more independently in the community. Most people accessing these services are diagnosed with schizophrenia and have complex care needs (Dalton-Locke et al., 2020; Parker et al., 2019a, 2019b, 2019c). Providing intensive support over an extended duration in a residential setting incurs high costs per episode of care (Parker et al., 2020). Despite the costs associated with these services, there have been limited comparative studies to establish their effectiveness (Dalton-Locke et al., 2020; Parker et al., 2019a, 2019b, 2019c), and TRR service capacity has expanded considerably in Australia over the last decade (Karan et al., 2022).

Australian TRR-type services have been adapted based on changing policy agendas and local service priorities. For example, services have generally shifted from a focus on providing a permanent residence to transitional support (Gerrand et al., 2007), increasingly emphasize recovery-oriented practice (McKenna et al., 2016), and are exploring novel staffing configurations with reduced emphasis on clinical roles (Karan et al., 2022; Parker et al., 2016; Saraf & Newton, 2017). Including staff with a lived experience of mental illness (Peer Support Workers, PSWs) in traditional clinical mental health services is increasingly encouraged in Australia (Saraf & Newton, 2017; State of Victoria, 2021). One such approach is the 'integrated staffing model' (Karan et al., 2022). Under this model, PSWs, rather than mental health nurses, represent the majority staffing component and draw on their personal recovery experiences to support consumers. The integrated staffing model was not intended to alter the principles and objectives of TRR care (Parker et al., 2016).

While there has been strong advocacy for the benefits of incorporating PSW roles in mental health services, the supporting evidence is mainly qualitative, with largely equivocal findings emerging from quantitative studies (Lloyd-Evans et al., 2014; O'Connor et al., 2017). Furthermore, there are concerns about how efforts to integrate paid PSWs within routine mental health care might undermine the value derived from 'real-world interactions between people supporting each other with their emotional distress' (p342) (Gillard, 2019).

Qualitative research conducted at TRR units trialing the integrated staffing model found that consumers and staff held positive expectations of this approach (Meurk, Parker, Newman, & Dark, 2018; Parker et al., 2018, 2019a, 2019b, 2019c). Furthermore, 12–18 months following service entry, consumers supported under this staffing approach emphasized the value of PSW availability (Parker et al., 2021). A recent cross-sectional study in Queensland found that the integrated staffing model was associated with lower levels of restrictive practice, pharmacological treatment, and greater staff-rated consumer engagement than the clinical staffing model (Karan et al., 2022). The authors of this study emphasized the need for findings to be interpreted with caution as no inferences could be drawn as to whether the differential treatment was appropriate or preferable. No available research compares care outcomes between the integrated and clinical staffing models. Despite the limited quantitative evidence, the integrated staffing model was identified as 'show[ing] promise in supporting recovery-oriented practice and maximizing consumer choice and control' (p37) in a report commissioned by the 2020 Victorian Royal Commission into Mental Health (Harvey & Brophy, 2020). Before the broader dissemination of such an approach, it is critical to demonstrate that a substantial reduction in clinical staff within TRR services to accommodate PSW availability does not degrade the clinical and functional outcomes achieved.

### Aims

This study considers whether clinical and functional outcomes differ between consumers admitted to community rehabilitation units operating the integrated and clinical staffing models. Given that the integrated staffing model was not intended to alter the core function of the service, we hypothesized that significant differences in outcomes between the staffing approaches would not emerge (Parker et al., 2016). However, if differences are present, this would affect the optimal staffing approach for future services.

### Methods

Data were collected as part of a mixed-methods evaluation of the comparative effectiveness of integrated and clinical staffing models for Community Care Units (CCUs; ethics approval HREC/14/QPAH/62) (Parker et al., 2016). A prospective observational design was used due to the ethical and clinical inappropriateness of randomized site allocation or waitlist control. Focusing on 'comparative effectiveness' followed the assumption of clinical equipoise at the policy level. Individualized change rather than group-level comparisons were chosen based on advocacy for this approach (Trauer, 2010) and its increasing use in similar contexts (Barbato et al., 2007; Gonda et al., 2012; Maxwell, Tsoutsoulis, Menon Tarur Padinjareveettil, Zivkovic, & Rogers, 2019; Murugesan et al., 2007). The ISPOR Task Force Report for comparative effectiveness research (Berger et al., 2012) and STROBE statement (von Elm et al., 2007) guided study reporting. Publications based on related data are available, including cohort description (Parker et al., 2019a, 2019b, 2019c), modelling predictors of unplanned discharge (Arnautovska et al., 2021), and qualitative research of stakeholder perspectives (Meurk et al., 2018; Parker et al., 2018, 2019, 2021).

### Study Context

CCUs are the dominant community-based TRR service-type operated by public mental health services in Australia. These units provide living skills development and community integration support to consumers residing in independent living units (in a cluster housing configuration) over 6-to-24-months. Staff support is available 24-h a day.

This study considered data from consumers admitted across three CCUs in Queensland over 3-years (12/2014-to-12/2017). One site operated the clinical staffing model, and two operated the integrated staffing model. Under the clinical staffing model, nursing staff reflect most staff roles; there are also senior allied health practitioners and medical staff. Under the integrated staffing model, PSWs reflect the majority team component (> 50%), with a reduction in the number of nursing roles. PSW staff have their own leadership structure and draw on their lived recovery experience to support consumers and guide clinical staff toward recovery-oriented practices. There is no specified interventional framework for PSWs at the CCUs. However, a qualitative study provides a rich description of what PSWs perceived their roles to be, emphasizing self-disclosure and connection through ‘shared engagement in everyday activities… providing authentic opportunities to support residents deal with their experiences and fears… [building] relationships and trust… [and] reducing shame and isolation' (p5) (Wyder et al., 2020).

### Participants

Consumers were admitted to the CCU closest to their most-recent principal residence and were included in the cohort if they provided consent and stayed beyond the assessment period (6-weeks, n = 145/161). Ninety-one percent and 89% of eligible clinical and integrated staffing model site consumers consented. Recruitment exceeded the target to achieve > 80% power to detect a 15% difference in the Health of the Nation Outcome Scales (HoNOS) (Wing et al., 1998) set for the parent evaluation (n ≥ 100) that was based on the treatment change observed in an Australian community residential step-up/down service (Siskind et al., 2013).

### Data Collection and Measures

Unblinded trained multi-disciplinary team members completed an assessment battery on admission and discharge. Diagnostic and demographic information was collected at admission, and treatment-related variables were collected at admission and discharge (see Table 1). Based on the literature, a range of known confounders relevant to understanding rehabilitation outcomes were available in our data set (see Supplementary Materials 1).

**Table 1:** Features of the study sites, including characteristics of the clinical and integrated staffing models. Adapted from Parker et al. (2016)(Parker et al., 2016)

		Site 1	Site 2	Site 3
Staffing ^a^	Model	Clinical	Integrated	Integrated
	Total FTE staff	21.6	24.5	18.4
	Total FTE peer-support staff	0.6	16	10.4
	Total FTE clinical staff	19.5	7.5	7
	FTE staff: Consumer ratio	1.08	1.23	1.15
	Peer support staff: Clinical staff ratio	.003	2.13	1.49
Location	Distance from state capital (km)	4.2	30.9	21.2
	Relative Socio-economic Disadvantage (2013) ^b^	90	83	46
Referring district	Population	588,475	143,628	287,517
	Acute inpatient services	Yes	Yes	Yes
	Inpatient rehabilitation beds	No	Yes	No
	Community mental services	Yes	Yes	Yes
	Transitional housing team	Yes	No	No
	Community-based rehabilitation team	Yes	No	Yes
	Mental health homelessness outreach team	Yes	No	Yes
Philosophy of care	Recovery-oriented	Yes	Yes	Yes
	Strengths-based	Yes	Yes	Yes
	Designated rehabilitation focus	Yes	Yes	Yes
	Voluntary engagement in rehabilitation ^c^	Yes	Yes	Yes
	Individualized care planning	Yes	Yes	Yes
	Transitional support	Yes	Yes	Yes
Built environment	Operational commencement	2012	2014	2014
	Maximum occupancy (consumers)	20	20	16
	Self-contained independent living units	20	20	15
	Disabled access units	1/20	1/20	1/15
	Shared recreation and leisure facilities	Yes	Yes	Yes
Treatment/support	Evidence based therapeutic group programmes ^d^	Yes	Yes	Yes
	Individual psychotherapy support ^e^	Yes	Yes	Yes
	Living skills support and development	Yes	Yes	Yes
	Peer support interventions and availability ^f^	Limited	Prominent	Prominent
	Structured leisure and physical activities	Yes	Yes	Yes

^a^Staffing profile as at 12/2014 (operational commencement of Sites 2 & 3), FTE = Full Time Equivalent staff

^b^Local Government Area (LGA) percentile rank of Relative Socio-economic Disadvantage in comparison to all other LGAs in Australia, higher number reflects lower levels of disadvantage (scale 0–100)

^c^Involuntary consumers are accepted, but with an explicit emphasis on voluntary engagement with available rehabilitation activities

^d^Group therapies include: CBT for Psychosis, Cognitive Remediation, and Social Cognition and Interaction Training

^e^Individual therapies include: Cognitive Behavior Therapy (CBT) and Motivational Interviewing

^f^Detailed illustration of the nature of peer support work in practice at the integrated staffing model units is available in Wyder et al. (2021) (Wyder et al., 2020)

The assessment battery included measures relevant to the real-world planning of rehabilitation care (Parker et al., 2016). These covered: functioning and disability (HoNOS, Social Functioning Scale (SFS) (Birchwood et al., 1990), Allen’s Cognitive Levels (ACL) (Velligan et al., 1998), and Life Skills Profile-16 (LSP-16) (Rosen et al., 2001); clinical symptoms [Brief Psychiatric Rating Scale (BPRS-18) (Flemenbaum & Zimmermann, 1973), Scale for the Assessment of Negative Symptoms (SANS) (Andreasen, 1982), and Alcohol Use Disorders Identification Test (AUDIT) (Babor, Higgins-Biddle, Saunders, & Monteiro, 2001)]; and, wellbeing/recovery (Mental Health Inventory (MHI-38) (Veit & Ware, 1983) and the Stages of Recovery Instrument (STORI-30) (Andresen et al., 2013). Measures were selected based on the availability of data to support their reliability and validity, as well as pragmatic considerations relevant to the service context (Parker et al., 2016).

### Defining Reliable and Clinically Significant Change

Difference scores (admission-discharge) and subsequent analyses were conducted on measures with > 50% of paired data. Where relevant, scores were transformed so that positive differences reflected improvement on all measures.

The reliable change index (RCI) was calculated using the Christensen and Mendoza formula (Christensen & Mendoza, 1986). The clinical significance of an individual discharge score was operationalized based on three cut-off methods (N. S. Jacobson & Truax, 1991): [Cut-off 1] More than 2 *SD*s from the dysfunctional population mean (i.e., cohort mean at admission); [Cut-off 2] Within 2 *SD*s of the functional population mean (i.e., normative data); and [Cut-off 3] Closer to the functional population mean than the dysfunctional population mean.

Normative data for Cut-offs 2 and 3 for HoNOS and LSP-16 came from a study of Australian individuals with a psychotic disorder accessing mental health services who had experienced at least one inpatient or emergency department admission within five years but none within six months (n = 114) (Maxwell et al., 2019). For BPRS-18 and SANS, normative data came from a study of community-dwelling individuals with clinically stable 'chronic schizophrenia' without admissions in the previous six months (n = 120) (Baynes et al., 2000). Relevant functional population data was not identifiable for the other measures; thus, only Cut-off 1 could be applied. Where skewed data limited the ability to interpret RCS based on Cut-off 1 meaningfully, RCS improvement was not considered (N. Jacobson et al., 1988).

Reliable and clinically significant (RCS) change was assumed where the change between admission and discharge score exceeded the RCI *and* crossed a clinical significance threshold (i.e., RCS improvement or RCS deterioration).

### Analysis

Analyses were completed in IBM SPSS Statistics Version 27 (SPSS, 2017). Comparability of the three sites was supported by examining measures at admission using the Kruskal–Wallis test (Supplementary Materials 2). Data from integrated staffing model sites were merged for subsequent analyses.

Individual difference scores were categorized as 'reliable improvement ‘or’ no reliable improvement' (i.e., stable/deterioration) based on the RCI. The RCS improvement cut-off producing the largest proportion of improved consumers (Gonda et al., 2012) was used to categorize scores into ‘improvement’ and ‘no improvement’ for RCS change. Outcomes for the staffing model groups were compared using Chi-Square/Fisher’s Exact tests, with effect size estimated using Cramer’s V (Kim, 2017).

The impact of known confounders on the relationship between staffing model and reliable improvement was explored using binomial logistic regression modelling (see Supplementary Materials 1). Modelling was not undertaken for RCS improvement due to the low event rates. Independent variables (IVs) additional to the ‘Integrated staffing model’ entered in the final models were rationalized based on a threshold of p < 0.200 (Mickey & Greenland, 1989). All IVs were entered simultaneously. Where time-related covariates were included, interactions with staffing model were explored. Events-to-IVs ratios in the final models exceeded the acceptable minimum threshold (5:1) in assessing confounders (Vittinghoff & McCulloch, 2007).

## Results

The sample included 145 consumers aged 18–59 years (M = 31.4, SD = 9.0, see Table 2). The median duration of CCU care was 303-days. Although there were no differences in the frequency of involuntary treatment on admission between the two staffing models, consumers under the integrated staffing model were more frequently voluntary at the time of discharge (χ2(1) = 4.061, p = 0.044). Paired admission and discharge data were available for > 50% of consumers on all measures except ACL and STORI-30 (Table 2).

**Table 2:** Characteristics of consumers by staffing model

	Clinical	Integrated	Total	Test statistic	p
	(n = 53)	(n = 92)	N = 145		
Demographics	–	–	–	–	–
Age at admission (x̅, years)	31.1 (8.7)	31.6 (9.2)	31.3 (9.0)	t_(143)_ = -0.318	0.751
Male sex	66.0%	78.3%	73.8%	χ^2^_(1)_ = 0.120	0.079
Australian born	86.8%	84.8%	85.5%	χ^2^_(1)_ = 0.811	0.472
Unemployment^a^	83.0%	90.2%	87.6%	χ^2^_(1)_ = 1.603	0.206
≤ 10-years formal education ^b^	47.2%	59.8%	54.5%	U = 2133.500	0.212
Accommodation (most recent) ^c^	–	–	–	Fisher's Exact	0.156
Living with family	59.8%	59.8%	58.6%	–	–
Income source ^d^	–	–	–	Fisher's Exact	0.104
Disability support pension	67.9%	54.3%	59.3%	–	–
Primary diagnosis	–	–	–	–	–
F20-29.x Schizophrenia spectrum	71.7%	80.4%	77.2%	χ^2^_(1)_ = 1.460	0.227
Secondary diagnoses/issues	–	–	–	–	–
Substance use	37.7%	48.9%	44.8%	χ^2^_(1)_ = 1.699	0.192
Physical health issue ^e^	22.6%	25.0%	24.1%	χ^2^_(1)_ = 0.841	0.457
Referral and treatment	–	–	–	–	–
Community-based referral ^f^	56.6%	63.0%	60.7%	χ2(1) = 0.585	0.445
Mean duration of CCU care (days) ^g^	402.5	329.6	356.3	U = 2295.000	0.557
Involuntary treatment ^h^ on admission	52.8%	43.5%	46.9%	χ2(1) = .1.181	0.277
Involuntary treatment at discharge	47.17%	30.43%	36.55%	χ2(1) = .4.061	0.044
More restrictive status	3.77%	2.17%	2.76%	Exact ^i^	0.609
No change in status	86.79%	82.61%	84.14%	–	–
Less restrictive status	9.43%	15.22%	13.10%	–	–

^a^Unemployment excludes any form of paid or unpaid form of employment but includes volunteering.

^b^Ordinal variable based on increasing levels of education with four categories (primary school, Year 10, Year 12, Tertiary), Mann-Whitney U test was applied. Tertiary education includes any vocational training regardless of completion.

^c^Six accommodation categories were considered: Living with family, Supported housing, Private rental, No fixed address, Other.

^d^Income source considered across three categories: Disability Support Pension, Other benefits (e.g. sickness benefits), Paid employment.

^e^Based on HoNOS item 5a ratings >2 being classified as a 'significant physical health issue'.

^f^Community-based referral compared to combined acute (35.2%) and sub-acute (4.1%) inpatient referral source.

^g^Range of CCU care (days) is 43-1361 (total), 43-1361 (clinical model), and 50-953 (integrated model).

^h^Involuntary treatment includes both Involuntary Treatment Orders/Treatment Authorities and Forensic Orders

^i^Comparing 'more restrictive status', 'no change in status', and 'less restrictive status' categories between the integrated and clinical staffing model groups for all participants, Exact = p=.609.

**Figure. 1:**
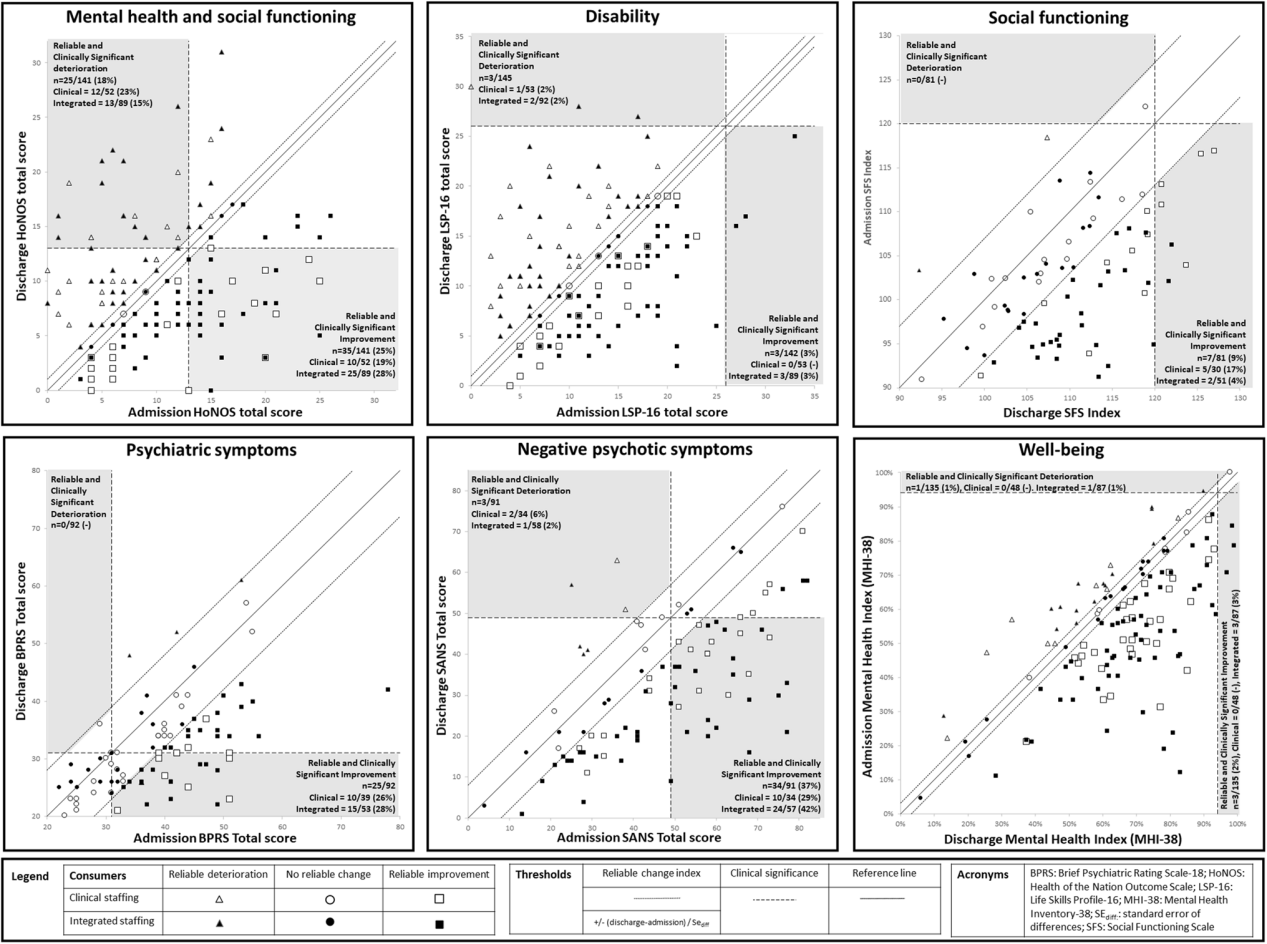
Plot of admission and discharge assessment scores for measures where reliable and clinically Significant change was calculable (deterioration / no change / improvement)

Most consumers showed reliable improvement on SANS (72.5%), MHI Index (64.0%), SFS (55.6%), LSP-16 (51.1%), and HoNOS (50.4%, see Table 3 and Fig. 1). Approximately half the consumers showed reliable improvement on BPRS-18 (47.8%). Using the RCS improvement criterion, gains occurred most frequently on the symptomatic measures (SANS, 37.4%; BPRS-18, 27.3%), and almost a quarter of participants improved on HoNOS (24.8%). Few consumers met the RCS improvement criterion on SFS (9.6%), MHI Index (2.2%), and LSP-16 (2.1%).

**Table 3:** Admission and discharge total scores on measures within the assessment battery where paired data was available for > 50% of participants (N = 145)

		Admission			Discharge		
	n	Mean (SD)	Median	Range	Mean (SD)	Median	Range
Functioning & disability							
HoNOS Total ^a^	141	10.3 (6.0)	9	0–26	9.8 (5.6)	9	0–31
* Clinical staffing*	52	9.1 (6.2)	7	0–25	9.2 (5.2)	9.5	0–23
* Integrated staffing*	89	11.1 (5.9)	10	0–22	10.1 (5.9)	9	0–31
LSP-16 Total ^a^	142	12.2 (6.1)	12	0–33	12.0 (6.1)	12	0–30
* Clinical staffing*	53	10.7 (5.7)	10	0–23	12.3 (6.0)	12	0–30
* Integrated staffing*	89	13.2 (6.2)	13	3–33	11.8 (6.1)	11	2–28
SFS Total (n = 81) ^b^	81	102.0 (7.5)	102.3	86–122	109.8 (7.6)	109.7	92–127
* Clinical staffing*	30	106.1 (7.7)	105.1	91–122	111.8 (8.7)	112.4	93–127
* Integrated staffing*	51	99.6 (6.3)	98.7	86–114	108.7 (6.8)	108.9	92–122
Symptoms							
BPRS-18 Total ^a^	92	39.0 (9.7)	39.5	22–78	31.67 (8.1)	30.5	18–61
* Clinical staffing*	39	37.4 (8.7)	39	23–55	30.9 (7.9)	31	20–57
* Integrated staffing*	53	40.3 (10.3)	41	22–78	32.2 (8.2)	30	18–61
SANS Total ^a^	91	47.6 (18.6)	49	4–82	33.6 (16.6)	33	1–76
* Clinical staffing*	34	39.8 (18.3)	36.5	4–76	28.7 (17.1)	24	1–66
* Integrated staffing*	57	52.3 (17.3)	53	14–82	36.6 (15.7)	37	4–76
Substance use							
AUDIT Total ^a^	78	6.4 (8.1)	3	0–32	5.4 (6.6)	3	0–28
* Clinical staffing*	27	4.5 (6.8)	2	0–22	3.2 (4.8)	1	0–23
* Integrated staffing*	51	7.4 (8.6)	3	0–32	6.5 (7.2)	3	0–28
Psychological well-being							
MHI Index ^b^	135	55% (18.9)	56.9%	5–100%	62.6% (18.7)	68.1%	6–99%
* Clinical staffing*	48	57.6% (17.1)	56.9%	21–100%	66.1% (18.0)	68.1%	14–98%
* Integrated staffing*	87	54.8% (19.9)	56.9%	5–95%	65.6% (19.1)	68.1%	6–99%

^a^Higher scores equate to higher levels of symptoms or impairment

^b^Lower score equate to higher levels of symptoms or impairment

In unadjusted analyses, only BPRS-18 scores differed between the two staffing models. A higher proportion of consumers admitted under the integrated staffing approach were reliably improved compared to the clinical staffing approach (60.4% vs. 30.8%, OR 3.43, CI 1.43–8.22, Cramer’s V 0.293). No group differences were statistically significant when the stricter RCS improvement criteria were applied.

Covariate analyses are detailed in Table 4. The increased likelihood of reliable improvement in BPRS-18 scores among consumers in the integrated staffing approach (Exp(B) = 2.726, CI = 0.994–7.480, p = 0.051) was attributable in part to baseline differences in HoNOS Total (Exp(B) = 1.090, CI = 1.009–1.177, p = 0.029) and education level (Exp(B) = 0.454, CI = 0.248–0.830, p = 0.010). After adjusting for confounders, consumers in the integrated staffing model also had higher odds of reliable improvement in SFS (Exp(B) = 3.218, CI = 1.122–9.229, p = 0.030). Additionally, analyses indicated the likelihood of RCI improvement was lower for consumers admitted later in the cohort for HoNOS (Exp(B) = 0.635, CI = 0.407–0.989, p = 0.045) and SANS (Exp(B) = 0.440, CI = 0.232–0.835, p = 0.012) Table 5 Table 4.

**Table 4:** Comparison of reliable and clinically significant improvement in outcome variables where > 50% of paired admission-to-discharge data was available

	Cut-off	Clinical n(%)	Integrated n(%)	Total n(%)	Test	Cramer's V ^c^	p
	Reliable improvement based on the Reliable Change Index (RCI) ^a^						
Functioning & disability							
HoNOS Total (n = 142)	−1	21(40.4)	50(56.2)	71(50.4)	χ^2^_(1)_ = 3.276	0.152	0.070
LSP-16 Total (n = 142)	−1	24(45.3)	50(56.2)	74(52.1)	χ^2^_(1)_ = 1.581	0.106	0.209
SFS Total (n = 81)	+ 7	13(43.3)	32(62.7)	45(55.6)	χ^2^_(1)_ = 2.883	0.189	0.090
Symptoms							
BPRS-18 Total (n = 91)	− 8	12(30.8)	32(60.4)	44(47.8)	χ^2^_(1)_ = 7.893	0.293	0.005
SANS Total (n = 91)	− 8	24(70.6)	42(73.7)	66(72.5)	χ^2^_(1)_ = 0.102	0.034	0.749
Substance use							
AUDIT Total (n = 78)	− 2	9(33.3)	18(35.3)	27(34.6)	χ^2^_(1)_ = 0.030	0.020	0.863
Psychological well-being							
MHI Index (n = 135)	+ 7	32(65.3)	55(63.2)	87(64.0)	χ^2^_(1)_ = 0.025	0.014	0.875

	Reliable and Clinically Significant (RCS) improvement						
Functioning and disability							
HoNOS Total (n = 142) ^a^	13	10 (19.2)	25 (28.1)	35 (24.8)	χ^2^_(1)_ = 1.380	0.099	0.240
LSP-16 Total (n = 142) ^a^	26	−(−)	3(3.4)	3 (2.1)	Fisher's Exact	–	0.293
SFS Total (n = 81)	120	5 (16.7)	2 (3.9)	7 (8.6)	Fisher's Exact	–	0.095
Symptoms							
BPRS Total (n = 91) ^a^	31	10 (25.6)	15 (28.3)	25 (27.2)	χ^2^_(1)_ = .080	0.030	0.777
SANS Total (n = 91) ^a^	49	10 (30.3)	24 (41.4)	34 (37.4)	χ^2^_(1)_ = 1.103	0.110	0.293
Psychological well-being							
MHI Index (n = 135) ^b^	94.1%	0 (0.0)	3 (3.4)	3 (2.2)	Fisher's Exact	–	0.552

^a^Normative population for the calculation of 'Cut-off 2' for HoNOS total and LSP-16 total was obtained from a study by Maxwell et al. (2018), and for the calculation of cut-off 3 for BPRS total, SANS total, and all SANS subscales from a study by Baynes et al. (2000)

^b^For the MHI, only RCS 'Cut-off 1' was calculable, the raw score cut off (215) is equivalent to a MHI percentage score of 94.1%. No RCS cut-off was able to be calculated for the AUDIT

^c^Effect size interpretation for Cramer’s V for chi-squared test with df = 1: 0.10 = small, 0.30 = medium, 0.50 = large

*M* mean; *SD* standard deviation; *RCI* reliable Change index; *RCS* reliable and clinically significant; *HoNOS* health of the nation outcome scales; *SFS* social functioning scale; *LSP*-16: life skills profile; *BPRS*-18: brief psychiatric rating scale; *SANS* scale for the assessment of negative symptoms; *AUDIT* alcohol use disorders identification test; MHI: mental health index

**Table 5:** Results of covariate analysis using logistic regression to identify predictors of reliable improvement

Outcome ^a^	Covariate(s) with p-value < 0.200 ^b,c,d^	Exp(B)	95% CI	p-value
AUDIT	Primary diagnosis of Schizophrenia	.515	0.191–1.392	0.191
	Comorbid substance use disorder	2.220	0.829–5.940	0.112
BPRS	Integrated staffing model	2.726	0.994–7.480	0.051
	Education level	.454	0.248–0.830	0.010
	Primary diagnosis of Schizophrenia	2.184	0.804–5.933	0.125
	HoNOS total	1.090	1.009–1.177	0.029
HoNOS	Admission date (years from study commencement)	.635	0.407–0.989	0.045
	CCU length of stay	1.692	0.922–3.106	0.090
	Integrated staffing model	1.984	0.802–4.911	0.138
	Male gender	2.707	0.989–7.408	0.052
	Primary diagnosis of Schizophrenia	.364	0.148–0.900	0.029
	Comorbid substance use disorder	.477	0.205–1.111	0.086
	HoNOS Total score	1.249	1.144–1.365	0.000
LSP-16	HoNOS Item 1 – Aggression	.642	0.386–1.068	0.088
	Life Skills Profile Total	1.212	1.122–1.309	0.000
MHI	Length of CCU stay	2.152	1.163–3.984	0.015
	HoNOS Item 1 – Aggression	1.475	0.864–2.519	0.155
SANS	Admission date (years from study commencement)	.440	0.232–0.835	0.012
	Education level	2.579	1.253–5.310	0.010
SFS	Integrated staffing model	3.218	1.122–9.229	0.030
	Comorbid substance use disorder	2.232	0.813–6.123	0.119
	LSP-16 Total score	.946	0.869–1.030	0.198
	Involuntary mental health act status	2.462	0.913–6.636	0.075

^a^Reliable improvement based on whether the difference between admission and discharge scores exceeded the calculated Reliable Change Index threshold

^b^Apart from the time-based variables ('Years between study commencement and admission' and 'Length of stay') all covariates are based on information at the time of admission

^c^Selection of covariates in addition to 'Integrated staffing model' was based on initial screening to identify available known confounders whose p-value was < .200. Covariates in the final models in were: Audit = Primary diagnosis F20.x, & Comorbid substance use); BPRS (Education level, Primary diagnosis F20.x, Comorbid substance use, & HoNOS Total score); HoNOS (Admission date, CCU length of stay, Gender, Primary diagnosis F20.x, Comorbid substance use, & HoNOS total score); LSP-16 (HoNOS Item 1 – Aggression, & LSP-16 total score); MHI (CCU length of stay, Gender, HoNOS Item 1 – Aggression, & HoNOS Item 5 – Physical); SANS (Admission date, & Education level); SFS (Comorbid substance use, LSP-16 total score, & Involuntary MHA status)

^d^Interactions with staffing model were considered for any outcome where time-based covariates ('Years between study commencement and admission' and 'Length of stay') had a p-value of < .200 in the final model. None of these interactions were included in the final models

## Discussion

This study considered whether consumers receiving TRR support under integrated and clinical staffing models achieve equivalent functional and clinical outcomes at discharge. Regardless of staffing model, most consumers (50.4%-72.5%) showed reliable improvements in negative psychotic symptoms (SANS), psychological wellbeing and distress (MHI-38), social functioning (SFS), disability (LSP-16), and mental health and social functioning (HoNOS). The unadjusted odds of reliable improvement were equivalent between the staffing model groups on all measures, except in general psychiatric symptoms (BPRS-18, favoring those in the integrated staffing model). No significant differences emerged in the likelihood of RCS improvement between the staffing model groups. Covariate analyses suggested that consumers admitted under the integrated staffing model were more likely to experience reliable improvement than in the clinical staffing model on two outcomes (BPRS-18 and SFS). Additional predictors of reliable improvement on the BPRS-18 were having a primary diagnosis of schizophrenia, higher HoNOS total score on admission, and lower levels of education. Additional predictors of reliable improvement in SFS emerging through the covariate analyses were the presence of comorbid substance use, involuntary mental health act status, and lower LSP-16 total scores on admission.

The gains in clinical and functional outcomes are consistent with the literature supporting the positive impact of mental health rehabilitation (Chan et al., 2021; Dalton-Locke et al., 2020). Unlike a recent retrospective cohort study that included only clinical staffing model sites (Parker et al., 2020), reliable improvements in disability occurred for most consumers. However, our study focused on admission-discharge outcomes rather than pre-admission and post-discharge. Our results also compare favorably to a recent Australian inpatient rehabilitation cohort study (Maxwell et al., 2019), with higher frequencies of reliable improvement in HoNOS and LSP. However, the comparability of these outcomes is limited by our mean length of admission being over three times longer.

The absence of marked differences between the integrated and clinical staffing configurations is consistent with the quantitative literature considering PSWs as care providers in Australian (O'Donnell et al., 1999) and international (Pitt et al., 2013) clinical services. Our findings indicate that the integrated staffing model achieved at least equivalent outcomes and that consumers under this model were more likely to have their involuntary treatment revoked prior to discharge. These findings provide reassurance that reduced restrictive and pharmacologically focused treatment at CCUs under the integrated staffing model (Karan et al., 2022) is not associated with inferior clinical and functional outcomes. Additionally, our findings add weight to the relevance of considering consumer preferences in terms of their emphasis on the value of the availability of PSWs under an integrated staffing model (Parker et al., 2021).

### Limitations

A key limitation is the absence of process evaluation to identify treatment differences between the approaches. Additionally, inter-rater reliability data was unavailable, and a later admission date was associated with a lower likelihood of reliable improvement on HoNOS and SANS, suggesting possible impacts of processes and staff changes. Unmeasured and unknown confounders may have also impacted the results. For example, service-level factors that impact organizational performance were not considered, such as staffing turnover, shortages, and burnout (Coates & Howe, 2015).

Outcomes were considered without correcting for multiple comparisons, increasing the risk of Type 1 error. The nature of the planned analyses meant that applying such corrections would have limited the ability to draw meaningful conclusions due to inflation of the Type 2 error risk (Armstrong, 2014). Another important consideration is limitations in statistical power to detect small differences between the staffing models. This is a particularly relevant consideration for the outcomes with higher levels of missing data (AUDIT, BPRS, SANS, and SFS). Given this, our findings should be interpreted cautiously.

Unavailability of paired admission data occurred more frequently for consumers who had experienced unplanned discharge. This means the findings may be biased toward consumers who are more likely to have favorable outcomes. Additionally, high rates of missing data prevented comparing the personal recovery measure (STORI-30), an outcome highly relevant to the service focus.

## Conclusions

Reliable improvements in symptoms and functioning generally occurred between admission to and discharge from community-based residential rehabilitation. Furthermore, most consumers demonstrated clinically significant improvements in negative psychotic symptoms and disability. Under the integrated and clinical staffing models, consumers had at least equivalent clinical and functional outcomes. In the context of other emerging research, our findings further emphasise the promising nature of the integrated approach as an alternative to traditional clinical staffing models. More research in other contexts will enhance the ability for future decisions about mental health rehabilitation services staffing to be evidence-informed.

## Supplementary Information

Below is the link to the electronic supplementary material.

Supplementary file1 (PDF 907 KB)

Supplementary file2 (PDF 250 KB)

## Data Availability

Any further release of data would be subject to application and approval from the relevant ethics committee and data custodians under the provisions of the Public Health Act (Queensland) 2005.
