# Supplementary material for: Comparative Effectiveness of Integrated Peer Support and Clinical Staffing Models for Community-Based Residential Mental Health Rehabilitation: A Prospective Observational Study
Source: Community Ment Health J. 2022 Sep 3;59(3):459–70. doi: 10.1007/s10597-022-01023-8 (PMC9981709; doi:10.1007/s10597-022-01023-8)
Supplement: Supplementary file 1 — Supplementary file1 (PDF 907 KB) [file 10597_2022_1023_MOESM1_ESM.pdf]

<u>Step 1:</u> Variables considered as outcomes and predictors for modelling

Outcome considerations

Outcome	Calculable	Event (E)	Non-event (NE)	Sample N
AUDIT-RCI improvement	Yes	27	51	78
AUDIT-RCS improvement	No	N/A	-	-
BPRS-RCI improvement	Yes	44	48	92
BRPS-RCS improvement	Yes	25	67	92
HoNOS-RCI improvement	Yes	71	70	141
HoNOS-RCS improvement	Yes	35	106	141
LSP-RCI improvement	Yes	74	68	142
LSP-RCS improvement	Yes	3	139	142
MHI-RCI improvement	Yes	87	48	135
MHI-RCS improvement	Yes	3	132	135
SANS-RCI improvement	Yes	66	25	91
SANS-RCS improvement	Yes	34	57	91
SFS-RCI improvement	Yes	45	36	81
SFS-RCS improvement	Yes	7	74	81

Audit RCS not able to be calculated.

Covariate considerations:

Type [a]	Predictor	Type	Rationale
Time and Service	Admission date [a]	Continuous	Variables related to the mental health service (e.g. staffing models and sites), and time based variables that may be associated with changes in the nature of service delivery. Hence this level address potential differences between the sites, and within he sites overtime relating to the service model. Note that the clinical site was not included as its own site variable as this site is separated from the integrated staffing model sites through the 'Staffing model' variable.
	CCU Length of stay [b]	Continuous	
	Staffing model	Dichotomous	
	Integrated site A	Dichotomous	
	Integrated site B	Dichotomous	
Consumer	Age (at admission date)	Continuous	Variables that reflecting largely static characteristics of the participants (i.e. well established and presumed to be stable prior to CCU admission). Note also that primary diagnosis differed significantly between the staffing model groups within our sample.
	Sex	Dichotomous	
	Education [c]	Scaled	
	Primary Dx F20-29.x [d]	Dichotomous	
	Comorbid substance use [e]	Dichotomous	
	Comorbid PD [f]	Dichotomous	
Impairment at admission	HoNOS admission total [g]	Continuous	Recent state related variables that are reflective of severity of mental health conditions described in Level 2.
	HoNOS admission Q1 (aggression) [h]	Scaled	
	HoNOS admission Q4 (cognition) [i]	Scaled	
	HoNOS admission Q5 (physical) [j]	Scaled	
	LSP-16 admission total [k]	Continuous	
	Involuntary status on admission	Dichotomous	

[a] Admission date (years from first integrated site commencement of operation (01/12/2014))
[b] Length of stay based on service defined admission and discharge data in years, inclusive of any leave periods
[c] Scaled variable of highest educational attainment: 1=Primary school, 2=10 years of formal education, 3=12 years of formal education, 4=Any tertiary education
[d] Formal diagnosis or working primary clinical diagnosis at the time of admission based on chart review
[e] Non-tobacco related substance use issues or history of current clinical relevance identified based on clinical documentation on admission or within the first 6-weeks of CCU care
[f] Formal diagnosis of a personality disorder or documentation of clinically significant personality disorder traits on admission or within the first 6-weeks of care based on chart review
[g] As a global measure of mental health and social functioning
[h] 'Overactive, aggressive, disruptive or agitated behaviour' rated on a scale of 0 ('no problem') to 4 ('severe or very severe problem')
[i] 'Cognitive problems' rated on a scale of 0 ('no problem') to 4 ('severe or very severe problem')
[j] 'Physical illness or disability problems' rated on a scale of 0 ('no problem') to 4 ('severe or very severe problem')
[k] As a global measure of disability
[l] Subject to an Involuntary Treatment Order or Forensic Order under the Queensland Mental Health Act (2000), or a Treatment Authority, Treatment Support Order, or Forensic Order under the Queensland Mental Health Act (2016) at the time of admission

Predictors and their organization is based on the approach adopted by Parker et al. (2020)[1]. The key deviations from the Parker et al. study being:

- Collapsing time and service model within a single level, as all relate to variables that may reflect changes between the sites (static or over time) due to site-based variation, staffing variation.
- Inclusion of 'Education level' – a known predictor of the rehabilitation outcomes that was not available in the Parker et al (2020) dataset.
- Co-morbid substance use (yes/no) being derived from a comprehensive chart review approach rather than relying on a single item from the HoNOS (Q3) as this was expected to have more complete and accurate information (note that this HoNOS item also informed the coding of the variable) and reduce the impact of collinearity given the use of the HoNOS Total score as a predictor.
- Shifting HoNOS items 1, 4, & 5 to impairment as HoNOS is not intended to reflect static characteristics of the consumer.
- Including involuntary status at the 'Impairment level' as all variables at this level are indicative of more immediate (i.e. state-based) features associated with illness
- The absence of availability of service utilization measures (ED presentations and mental health bed days) as these were not present in the data set

Note that the included variables and approach in this study partially differ from the planned predictors outlined in the parent study protocol [2]. These deviations have followed a more comprehensive review of recent literature to identify relevant predictors/covariates, availability of additional studies to guide covariate selection, and the implementation experience of data collection for the cohort (including levels of missing data affecting several variables).

Evidence supporting chosen covariates: *

* This table is an adaptation of Table 2 in the Supplementary Materials of the Parker et al. (2020) modelling study

Study	Sample	Context and country	Design	Outcome measures	Independent predictor variables	Main findings
Chatterjee et al. (2009)[3]	236 patients (141 males)<br>Age: 20-45 years (74.2%)<br>Primary diagnoses: Schizophrenia (55.5%), bipolar (27.7%), and other psychoses (16.8%)	Community-based rehabilitation in rural India; minimum enrollment in the program was 12 months with a median period of 46 months	Longitudinal with assessments at admission and discharge over a 3-year follow-up period	Disability assessed with Indian Disability Evaluation Assessment Scale (IDEAS)	Marital status, primary diagnosis, duration of illness, medication adherence, household assets, family support, self-help group membership, program drop-out	Improvement was marked (≥ 40% change from baseline) in 50%, moderate (20-40% change from baseline) in 40%, and minimal (<20% change from baseline) in 10% of participants<br>Positive predictors of reduced levels of disability were lower baseline disability, family engagement with the program, medication adherence, and engagement with a self-help group; negative predictors were lack of formal education, diagnosis of schizophrenia and dropping out of the program
De Girolamo et al. (2014)[4]	403 patients (2/3 male)<br>Mean age: 48 ± 10, range 19-64 years<br>Primary diagnoses: Schizophrenia-spectrum (67.5%) and personality disorders (17.9%)	23 medium-long-term residential facilities in Italy; mean length of stay: 4.2 years ± 5.5 (median = 2.2)	Longitudinal with 1-year follow-up	Likelihood of home discharge	Socio-demographic (e.g., primary diagnosis, illness duration, age), psychosocial variables (e.g., social support, inactivity)	Positive predictors of home discharge were shorter illness duration, available social support in the last year, and a diagnosis of unipolar depression
Gonda et al. (2012)[5]	337 patients (170 male)<br>Mean age: 33.6 ± 9.8, range 18-61 years<br>Primary diagnoses: Schizophrenia (68%) and schizoaffective (20.8%) disorders	2 inpatient psychosocial rehabilitation units in NSW, Australia; mean length of stay: 111 ± 73, range 6-602 days	Longitudinal with assessments at admission, discharge and 3-month follow-up	RCS improvement on BPRS-E, HoNOS, and K10 (RCS improved / not improved)	Age, gender, length of stay, primary diagnosis and co-morbid diagnosis	Between 32% and 49% of the patients made an improvement; between 20% and 32% made RCS improvement across the three outcome measures<br>Positive predictors of RCS improvement on psychiatric symptomatology (BPRS-E) were schizoaffective disorder [exp(β) = 3.52, $p < 0.05$] and co-morbid alcohol abuse disorder [exp(β) = 2.29, $p = 0.053$]
Grinshpoon et al. (2007)[6]	4160 patients (2413 male) discharged from their first-in-life psychiatric hospitalization<br>Primary diagnoses: F20-F29 or F30-F39 (100%)	Two cohorts of patients (discharged 1990-1991 and 2000-2001), to assess the effects of the Rehabilitation of the Mentally Disabled Act (RMDA) passed in 2000, Israel	Longitudinal with 3-year follow-up	Time to hospital re-admission	Age, gender	Re-admission for all patients was lower among females [exp(β) = 0.13, $p < 0.001$] and negatively associated with age [exp(β) for 20-44 y old = 0.13, $p = 0.001$ and for 45-64 y old = 0.414, $p < 0.001$]<br>For the 2000-2001 cohort, long hospitalization (more than 6 months) was associated with reduced likelihood of re-admission during follow-up [exp(β) = 0.26, $p = 0.056$]

Study	Sample	Context and country	Design	Outcome measures	Independent predictor variables	Main findings
Killaspy and Zis (2013)[7]	141 patients (84 male)<br>Mean age: 44 ± 13 years<br>Primary diagnoses: Schizophrenia or schizoaffective disorder (93%)	Mental health residential rehabilitation service in London, UK, incl. 2 shorter-term inpatient units (n = 47), 3 community units (n = 44), 4 community-supported accommodation facilities (n =50)	Retrospective 5-year survey-based study	Change in independence: positive outcome - achieving and sustaining community placement for inpatients and progressing or sustaining a less supported community placement for community patients (progressed / remained stable / relapsed)	Age, history of physical abuse, medication non-adherence, challenging behaviours, social function communication, involuntary treatment	Positive outcome was achieved by 50 (40%) of the patients; 13 (10%) moved to independent accommodation and successfully sustained their tenancy; 33 (27%) remained in a placement with a similar level of support; 41 (38%) moved to more supported placement and/or had a psychiatric admission<br>Increased age was associated with a reduced likelihood (OR = 0.93, $p = 0.003$) and medication non-adherence with increased odds of a negative outcome (OR = 33.57, $p < 0.001$)
Lim et al. (2017)[8]	246 patients (161 male)<br>Mean age: 37.9 ± 9.4 years<br>Primary diagnosis: Schizophrenia (100%)	6 community-based psychiatric rehabilitation programs in Los Angeles, USA	Longitudinal with assessments at admission, and at 6 and 12 months after admission	Recovery based on 4 criteria (be in symptomatic remission, demonstrate adequate work and social functioning, and no psychiatric hospitalization)	Demographic (gender, education), clinical (e.g., symptomatology, length of illness, medication use), and psychosocial characteristics (e.g., intrinsic motivation, social support)	Recovery was recorded in 19.8% and 7.5% of patients at 6- and 12-month follow-up, respectively (n = 146)<br>Higher levels of intrinsic motivation [exp(β) = 1.68], positive family relationships [exp(β) = 1.32], role functioning [exp(β) = 1.34], and social functioning [exp(β) = 1.40] at admission predicted recovery at 6-month follow-up (all $p < 0.05$)
Maxwell et al. (2018)[9]	Clinical group: 210 patients (144 male)<br>Comparison group (mental health sample functioning independently in the community): 114 adults (57 male)<br>Primary diagnosis: Schizophrenia (100%)	1 inpatient mental health rehabilitation unit in NSW, Australia	Longitudinal with assessments at admission, discharge and at least 1 year post-discharge	RCS (based on cut-off 3) on HoNOS; LSP-16; and K10	Age, gender, marital status, type of usual accommodation, country of birth, secondary diagnosis, length of stay, HoNOS total and subscale scores and LSP total and subscale scores at admission	Positive predictors of RCS improvement on HoNOS total scale were HoNOS Behaviour [exp(β) = 14.57 $p < 0.01$] and Impairment subscales scores [exp(β) = 18.87, $p < 0.05$] at admission<br>Positive predictors of RCS improvement on LSP total scale were LSP Socialisation [exp(β) = 10.23, $p < 0.05$] and Withdrawal subscales scores [exp(β) = 10.23, $p < 0.05$] at admission

Study	Sample	Context and country	Design	Outcome measures	Independent predictor variables	Main findings
Parker et al. (2020)[1]	N=501 complete sampling, predominant diagnoses of F20-29.x disorders.	Statewide rehabilitation outcomes of 512 consumers at Qld CCUs over 2015-2014.	Retrospective cohort study, secondary analysis of routine administrative data.	Multiple: change in HoNOS and LSP-16, mental health service utilization, involuntary treatment.	Year of admission, Site, Sex, Age, Primary diagnosis, Personality disorder (secondary), HoNOS and LSP (including aggression, substance use, cognitive impairment and physical health items), bed-based service use, ED presentations, family involvement, involuntary status at entry	Significant predictors of improvement included variables related to the CCU care (e.g. episode duration), consumer characteristics (e.g. primary diagnosis) and treatment variables (e.g. psychiatry-related bed days pre-admission). Higher baseline impairment in mental health and social functioning (β = 1.12) and longer episodes of CCU care (β = 1.03) increased the likelihood of RCS improvement in mental health and social functioning.
Yoon et al. (2013)[10]	9208 adults<br>Mean age: 41.1 ± 15.6 years<br>Diagnoses: Schizophrenia (63%), bipolar (48%), other mental illness (52%), substance abuse (54%)	Intensive case-management community-treatment program, California, USA; mean tenure: 10.8 ± 8.2 months	Longitudinal followed up to 4 years	Residential transition to different types of living arrangements	Length and continuity of program participation, age, gender, diagnosis, education, race	Positive predictors of independent living arrangement were uninterrupted program participation, having a diagnosis of bipolar disorder (relative to schizophrenia), and any other diagnosis, such as depression or personality or anxiety disorder (relative to schizophrenia or bipolar disorder)

BPRS-E: Brief Psychiatric Rating Scale-Expanded version; HoNOS: Health of the Nation Outcome Scales; K10: Kessler 10; LSP: Life Skills Profile-16; NSW: New South Wales; OR: adjusted odds ratio;

RCS: Reliable and clinically significant; SLOF: Specific Levels of Functioning; UK: United Kingdom; USA: United States of America

<u>Step 2:</u> Is logistic regression appropriate based on the planned number of potential predictors and the event rates?

Outcome considerations

Outcome	Calculable	Event (E)	Non-event (NE)	Sample N	Lower of N or NE /10	Lower of N or NE / 5	Outliers	Include	Rationale
AUDIT-RCI improvement	Yes	27	51	78	2.7	5.4	0	Yes	
AUDIT-RCS improvement	No	N/A	-	-	-	-	-	No	Unable to be calculated
BPRS-RCI improvement	Yes	44	48	92	4.4	8.8	0	Yes	
BRPS-RCS improvement	Yes	25	67	92	2.5	5	0	Yes	Unacceptably high risk of overfitting [a]
HoNOS-RCI improvement	Yes	71	70	141	7	14	0	Yes	
HoNOS-RCS improvement	Yes	35	106	141	3.5	7	0	Yes	Unacceptably high risk of overfitting [a]
LSP-RCI improvement	Yes	74	68	142	6.8	13.6	1	Yes	
LSP-RCS improvement	Yes	3	139	142	.3	.6	1	No	Unacceptably high risk of overfitting [a]
MHI-RCI improvement	Yes	87	48	135	4.8	9.6	0	Yes	
MHI-RCS improvement	Yes	3	132	135	.3	.6	0	No	Unacceptably high risk of overfitting [a]
SANS-RCI improvement	Yes	66	25	91	2.5	5	1	Yes	
SANS-RCS improvement	Yes	34	57	91	3.4	6.8	1	Yes	Unacceptably high risk of overfitting [a]
SFS-RCI improvement	Yes	45	36	81	3.6	7.1	0	Yes	
SFS-RCS improvement	Yes	7	74	81	.7	1.4	0	No	Unacceptably high risk of overfitting [a]

[a] The decision was made to omit the RCS outcome from the covariate analysis given the low event rate relative to RCI, and that related RCI analyses considered similar outcomes

Outcome selection for the confounder/covariate analysis was guided by the high number of potential confounders identified in the literature and available in our dataset (n=17) relative to the available sample size (n=145, with variable event rates and levels of missing data across the variables). The traditional rule of thumb of 10-events per IV in logistic regression was considered inappropriate in the context of the primary goal of the secondary analysis which was to identify the contribution of known confounders on the observed outcome, hence the less conservative target of 5 events per IV was set[11].

We had both RCI and RCS for most variables, with the event rates for RCS being substantially lower than RCI [as this is a more stringent outcome criterion]. The decision was made to limit the analysis to the RCI outcome only given the high risk of overfitting associated with low event rates.

After excluding the RCS outcome considerations, examination of event rates indicated that inclusion of up to 10-IVs would exceed the 5 (events) : 1 (predictor) ratio for two outcomes (HoNOS RCI and LSP MHI) and approximate this ratio for one outcome (MHI RCI). Emphasis on appropriate caution in interpreting the analyses for the remaining outcomes considered is needed (Audit RCI, SANS RCI, and SFS RCI).

<u>Step 3:</u> Do the assumptions for binomal logistic regression hold?

Absence of collinearity:

- Assessment of correlation matrix completed, Spearman's correlation (non-parametric) not >.7, robust for all. However, variables of potential concern (>.4) were:
- Staffing model and each of the Integrated site variables (Bayside site=.568, Logan site=.468). This intercorrelation was expected given that the Staffing model variable contained only participants from these sites. The decision was made to drop the Logan site variable as this contained the smaller number of participants, and both site variables were not needed to consider site-based variation between the integrated sites.
- HoNOS Total as expected was correlated with the single items from this measure that were also included: Item 4 – Cognition (.557) > Item 1 – Aggression (.516) > Item 5 – Physical (.369). However, the correlations between the individual HoNOS items were low. The decision was made to omit HoNOS Item 4 – Cognition, which had the highest correlation with the total HoNOS score, from the potential predictors.

Correlations

			Years between admission date and cohort inception	Staffing_model	Bayside_site_dummy	Logan_site_dummy	LOS_years	Consumer Age at commencement (years)	Gender	Education level at admission	F20-29 at admission	Comorbid Substance use disorder (current, not tobacco) at admission	Comorbid Personality Disorder at admission	HoNOS Behaviour Q1 Overactive, aggressive, disruptive or agitated at admission	HoNOS Impairment Q5 Physical illness or disability problems at admission	HoNOS Impairment Q4 Cognitive problems at admission	HoNOS_total_Admit	LSP_total_Admit
Spearman's rho	Staffing_model	Correlation Coefficient	-.084															
		Sig. (2-tailed)	.317															
		N	145															
	Bayside_site_dummy	Correlation Coefficient	-.065	.568														
		Sig. (2-tailed)	.439	<.001														
		N	145	145														
	Logan_site_dummy	Correlation Coefficient	-.021	.468	-.462													
		Sig. (2-tailed)	.805	<.001	<.001													
		N	145	145	145													
	LOS_years	Correlation Coefficient	-.087	-.049	-.125	.081												
		Sig. (2-tailed)	.299	.559	.134	.330												
		N	145	145	145	145												
	Consumer Age at commencement (years)	Correlation Coefficient	-.130	.023	.075	-.055	-.022											
		Sig. (2-tailed)	.118	.779	.370	.510	.796											
		N	145	145	145	145	145											
	Gender	Correlation Coefficient	.059	.134	.086	.052	.100	.027										
		Sig. (2-tailed)	.481	.108	.304	.534	.231	.747										
		N	145	145	145	145	145	145										
	Education level at admission	Correlation Coefficient	-.037	-.113	-.046	-.072	.038	.018	.030									
		Sig. (2-tailed)	.656	.177	.582	.389	.648	.829	.717									
		N	145	145	145	145	145	145	145									
	F20-29 at admission	Correlation Coefficient	-.025	.100	-.074	.188	.065	.006	.125	-.034								
		Sig. (2-tailed)	.762	.230	.375	.024	.436	.942	.133	.688								
		N	145	145	145	145	145	145	145	145								
	Comorbid Substance use disorder (current, not tobacco) at admission	Correlation Coefficient	.002	-.108	-.136	.029	.208	.066	-.064	.165	-.026							
		Sig. (2-tailed)	.981	.195	.104	.730	.012	.429	.443	.048	.754							
		N	145	145	145	145	145	145	145	145	145							
	Comorbid Personality Disorder at admission	Correlation Coefficient	.005	-.037	-.080	.046	-.041	.033	.209	.066	.047	.083						
		Sig. (2-tailed)	.957	.658	.337	.581	.625	.692	.012	.432	.574	.321						
		N	145	145	145	145	145	145	145	145	145	145						
	HoNOS Behaviour Q1 Overactive, aggressive, disruptive or agitated at admission	Correlation Coefficient	-.132	.068	-.046	.122	-.080	-.178	-.101	-.152	-.191	-.080	-.021					
		Sig. (2-tailed)	.116	.418	.589	.148	.345	.033	.231	.070	.022	.345	.802					
		N	143	143	143	143	143	143	143	143	143	143	143					
	HoNOS Impairment Q5 Physical illness or disability problems at admission	Correlation Coefficient	-.089	.049	-.109	.169	-.088	.133	-.164	-.146	-.093	.016	.020	.104				
		Sig. (2-tailed)	.289	.558	.195	.043	.295	.113	.050	.081	.270	.849	.813	.219				
		N	143	143	143	143	143	143	143	143	143	143	143	142				
	HoNOS Impairment Q4 Cognitive problems at admission	Correlation Coefficient	-.143	.206	-.027	.250	-.005	.013	-.008	-.166	-.038	.054	.060	.175	.062			
		Sig. (2-tailed)	.090	.014	.752	.003	.951	.877	.925	.049	.654	.526	.478	.038	.468			
		N	141	141	141	141	141	141	141	141	141	141	141	140	140			
	HoNOS_total_Admit	Correlation Coefficient	-.069	.202	-.017	.235	-.045	-.002	-.024	-.171	-.201	-.057	-.042	.516	.369	.557		
		Sig. (2-tailed)	.408	.015	.843	.005	.589	.985	.779	.041	.016	.499	.619	<.001	<.001	<.001		
		N	144	144	144	144	144	144	144	144	144	144	144	143	143	141		
	LSP_total_Admit	Correlation Coefficient	-.095	.184	.020	.177	.056	.029	-.045	-.187	.038	.051	.017	.270	.114	.423	.589	
		Sig. (2-tailed)	.257	.028	.814	.034	.503	.728	.596	.025	.651	.544	.842	.001	.175	<.001	<.001	
		N	144	144	144	144	144	144	144	144	144	144	144	143	143	141	144	
	Involuntary patient under MHA at admission	Correlation Coefficient	-.081	-.090	.075	-.178	-.136	.079	-.068	-.105	.115	-.153	-.017	.017	-.227	.018	-.091	.086
		Sig. (2-tailed)	.334	.280	.368	.032	.104	.343	.413	.209	.170	.066	.840	.845	.006	.833	.276	.307
		N	145	145	145	145	145	145	145	145	145	145	145	143	143	141	144	144

The reduced IV set was then assessed based on the Variance Inflation Factor (VIF) and Tolerance statistic for linear regression modelling of each of the RCI outcomes. Statistics across all to be included outcomes assessed to identify any variable with a VIF >10 or tolerance score below 0.2 [12]. Nil variables exceeded these thresholds.

		AUDIT		BPRS		HoNOS		LSP		MHI		SANS		SFS	
		Collinearity Statistics		Collinearity Statistics		Collinearity Statistics		Collinearity Statistics		Collinearity Statistics		Collinearity Statistics		Collinearity Statistics	
Model		Tolerance	VIF	Tolerance	VIF	Tolerance	VIF	Tolerance	VIF	Tolerance	VIF	Tolerance	VIF	Tolerance	VIF
1	Years between admission date and cohort inception	0.749	1.335	0.701	1.427	0.920	1.087	0.921	1.086	0.905	1.105	0.712	1.404	0.769	1.301
	Bayside_site_dummy	0.558	1.793	0.686	1.458	0.683	1.465	0.690	1.448	0.689	1.452	0.613	1.632	0.477	2.097
	LOS_years	0.718	1.393	0.874	1.144	0.886	1.129	0.882	1.133	0.883	1.132	0.796	1.257	0.752	1.329
	Consumer Age at commencement (years)	0.790	1.266	0.834	1.199	0.898	1.114	0.900	1.112	0.902	1.109	0.825	1.213	0.890	1.123
	Gender	0.792	1.262	0.804	1.244	0.838	1.193	0.840	1.191	0.844	1.185	0.791	1.265	0.771	1.298
	Education level at admission	0.762	1.313	0.814	1.229	0.895	1.117	0.894	1.119	0.896	1.116	0.827	1.210	0.827	1.209
	F20-29 at admission	0.769	1.301	0.780	1.282	0.795	1.257	0.794	1.259	0.789	1.267	0.720	1.388	0.837	1.195
	Comorbid Substance use disorder (current, not tobacco) at admission	0.765	1.307	0.840	1.191	0.850	1.177	0.849	1.178	0.842	1.188	0.820	1.220	0.795	1.258
	Comorbid Personality Disorder at admission	0.834	1.199	0.882	1.134	0.917	1.091	0.916	1.092	0.916	1.092	0.868	1.152	0.823	1.215
	HoNOS_total_Admit	0.262	3.813	0.288	3.472	0.318	3.147	0.316	3.164	0.299	3.343	0.276	3.623	0.289	3.460
	HoNOS Behaviour Q1 Overactive, aggressive, disruptive or agitated at admission	0.460	2.175	0.591	1.691	0.588	1.702	0.587	1.705	0.592	1.689	0.511	1.959	0.638	1.568
	HoNOS Impairment Q5 Physical illness or disability problems at admission	0.597	1.674	0.625	1.601	0.678	1.476	0.675	1.481	0.686	1.457	0.613	1.630	0.578	1.729
	LSP_total_Admit	0.550	1.818	0.479	2.089	0.536	1.865	0.536	1.866	0.504	1.982	0.510	1.959	0.470	2.126
	Involuntary patient under MHA at admission	0.732	1.366	0.700	1.429	0.814	1.228	0.814	1.228	0.813	1.231	0.693	1.444	0.774	1.292

Absence of outliers [continuous variables only: Age, Date of Admission, HoNOS Total, LSP Total]:

- AUDIT-RCI – no outliers with probability <.001 based on Mahalanobis distance
- BPRS-RCI – no outliers with probability <.001 based on Mahalanobis distance
- HoNOS-RCI – no outliers with probability <.001 based on Mahalanobis distance
- LSP-RCI – no outliers with probability <.001 based on Mahalanobis distance
- MHI-RCI – no outliers with probability <.001 based on Mahalanobis distance
- SANS-RCI – no outliers with probability <.001 based on Mahalanobis distance
- SFS-RCI – no outliers with probability <.001 based on Mahalanobis distance

Absence of continuous independent variables linearly related to the log odds

- Interactions between Admission date, Age, HoNOS total, LSP Total, and LOS with their log were checked within regression models with nil issues identified

<u>Step 4.</u> **Rationalizing potential covariates based on a p<.20 decision rule[13] across the entire variable set and a target of >10 cases per IV**

Type	Predictor	Type	AUDIT	BPRS	HoNOS	LSP	MHI	SANS	SFS						
			RCI	RCI	RCI	RCI	RCI	RCI	RCI	***	**	*	^	~	Total
Time & Service	Admission date	Continuous			*			*				2			2
	CCU Length of stay (years)	Continuous	~		^		*					1	1	1	3
	Staffing model	Dichotomous		*	~				*			2		1	3
	Integrated site A	Dichotomous		~										1	1
Consumer	Age	Continuous	~					~						2	2
	Gender	Dichotomous			~									1	1
	Education	Scaled		*				*	~			2			2
	Primary Dx F20-29.x	Dichotomous		^	*	~						1	1	1	3
	Comorbid substance use	Dichotomous	*		~				~			1		2	3
	Comorbid PD	Dichotomous	*					~	~			1		2	3
Impairment at admission	HoNOS total	Continuous		~	**		~				1			2	3
	HoNOS Q1 (aggression)	Scaled				^	^						2		2
	HoNOS Q5 (physical)	Scaled	~				^						1	1	2
	LSP-16 total	Continuous				***			~	1				1	2
	Involuntary MHA status	Dichotomous							~					1	1

~ p<.200-.100
^ p<.100-.050
* p<.050-.010
** p<.010-001
*** p<.001

Option A: Consistency of covariates across the outcomes

Given the large number of known potential confounders available in the data set relative to the sample size a target of 10 covariates was established (see Step 2). Decisions about which variables to include in the final analysis was guided by the following decision tree (with the aim of stopping when the target of ≤10 IVs was reached):

	Action	Rationale	Covariates		
			Start	End	Excluded
1	Include 'Staffing model' regardless of p-value	This is the IV of primary interest	15	15	N/A
2	Include any Level 1 (Time & Service) variable with p<.200 for an outcome variable	These variables are linked to the primary focus of the study and are relevant to understanding whether the impact of these organizational factors on the relationship between staffing model and outcomes (e.g. change in models over time, site-based variation between integrated sites).	15	15	N/A
3	Exclude any variable with only p>.200-.100 for a single IV across all outcomes	Limiting covariate inclusion to those with less predictive value than other available variables	15	13	Integrated site B<br>Involuntary MHA status
4	Exclude any variable with IVs only achieving p>.100 across all outcome variables.	Limiting covariate inclusion to those with less predictive value than other available variables	13	9	Age<br>Gender<br>HoNOS Q1<br>HoNOS Q5

Type	Predictor	Type	AUDIT	BPRS	HoNOS	LSP	MHI	SANS	SFS						
			RCI	RCI	RCI	RCI	RCI	RCI	RCI	***	**	*	^	~	Total
Time & Service	Admission date	Continuous			*			*				2			2
	CCU Length of stay (years)	Continuous	~		^		*					1	1	1	3
	Staffing model	Dichotomous		*	~				*			2		1	3
Consumer	Education	Scaled		*				*	~			2			2
	Primary Dx F20-29.x	Dichotomous		^	*	~						1	1	1	3
	Comorbid substance use	Dichotomous	*		~				~			1		2	3
	Comorbid PD	Dichotomous	*					~	~			1		2	3
Impairment at admission	HoNOS total	Continuous		~	**		~				1			2	3
	LSP-16 total	Continuous				***			~	1				1	2

~ p<.200-.100
^ p<.100-.050
* p<.050-.010
** p<.010-001
*** p<.001

The issue with this approach is that it results in 9 IVs for each outcome, many of which lacking clear predictive value across the outcome set.

Option B: Focused covariates for each outcome

	AUDIT	BPRS	HoNOS	LSP	MHI	SANS	SFS
	RCI	RCI	RCI	RCI	RCI	RCI	RCI
IV 1	Staffing model	Staffing model	Staffing model	Staffing model	Staffing model	Staffing model	Staffing model
IV 2	CCU Length of stay (years)	Integrated site A	Admission date	Primary Dx F20-29.x	CCU Length of stay (years)	Admission date	Education
IV 3	Age	Education	CCU Length of stay (years)	HoNOS Q1 (aggression)	HoNOS total	Age	Comorbid substance use
IV 4	Comorbid substance use	Primary Dx F20-29.x	Gender	LSP-16 total	HoNOS Q1 (aggression)	Education	Comorbid PD
IV 5	Comorbid PD	HoNOS total	Primary Dx F20-29.x		HoNOS Q5 (physical)	Comorbid PD	LSP-16 total
IV 6	HoNOS Q5 (physical)		Comorbid substance use				Involuntary MHA status
IV 7			HoNOS total				
IVs selected	5	5	7	3	4	4	6
Staffing added	Yes	No	No	Yes	Yes	Yes	No
Final IV count	6	5	7	4	5	5	6
Event rate (min)	27	44	70	68	48	25	36
Events / IV	4.5	8.8	10	17	9.6	5	6

- Staffing model is included in the modelling of all outcome given this is the principal variable from which we are interested in the impact of covariates.
- This approach focuses the modelling on the covariates most relevant to each outcome, and reduces the number of the covariates to the point where the minimum 5 events per IV threshold is exceeded for 5 of the 6 outcomes, and the desirable target of at least 10 events per IV is exceeded for two of these five outcomes.
- However, this approach loses consistency across variables and a total of 13 separate IVs are considered across the outcomes.

Step 4a. Using F20.x rather than F20-29.x

Final modelling solution for BPRS under the original approach resulted in very broad Exp(*B)* confidence intervals for ‘Primary diagnosis F20-29.x’ hence an alternative modelling approach where the diagnostic variable F20.x (yes/no) was considered. Note that ‘Integrated Site A’ was also removed from the covariate list based on the issues emerging during the previous modelling round when both this and the related variable (and principal IV focus) ‘Integrated Staffing Model’ had been included.

Type	Predictor	Type	AUDIT	BPRS	HoNOS	LSP	MHI	SANS	SFS						
			RCI	RCI	RCI	RCI	RCI	RCI	RCI	***	**	*	^	~	Total
Time & Service	Admission date	Continuous			*			*				2			2
	CCU Length of stay (years)	Continuous			^		*					1	1		2
	Staffing model	Dichotomous	~	*	^				*			2	1	1	4
Consumer	Age	Continuous						~						1	1
	Gender	Dichotomous	~		~		~							3	3
	Education	Scaled		*				*	~			2		1	3
	Primary Dx F20	Dichotomous	^	~	*							1	1	1	3
	Comorbid substance use	Dichotomous	~		^				~			1		2	3
	Comorbid PD	Dichotomous	*					~	~			1		2	3
Impairment at admission	HoNOS total	Continuous		~	***		^				1		1	1	3
	HoNOS Q1 (aggression)	Scaled				^	^						2		2
	HoNOS Q5 (physical)	Scaled	~	~			^						1	2	3
	LSP-16 total	Continuous				***			^	1				1	2
	Involuntary MHA status	Dichotomous							~					1	1

~ p<.200-.100
^ p<.100-.050
* p<.050-.010
** p<.010-001
*** p<.001

Step 4a - Option A: Consistency of covariates across the outcomes

Given the large number of known potential confounders available in the data set relative to the sample size a target of 10 covariates was established (see Step 2). Decisions about which variables to include in the final analysis was guided by the following decision tree (with the aim of stopping when the target of ≤10 IVs was reached):

	Action	Rationale	Covariates		
			**Start**	**End**	**Excluded**
1	Include 'Staffing model' regardless of p-value	This is the IV of primary interest	14	14	N/A
2	Include any Level 1 (Time & Service) variable with p<.200 for an outcome variable	These variables are linked to the primary focus of the study and are relevant to understanding whether the impact of these organizational factors on the relationship between staffing model and outcomes (e.g. change in models over time, site-based variation between integrated sites).	14	14	N/A
3	Exclude any variable with only p<.200-.100 for a single IV across all outcomes	Limiting covariate inclusion to those with less predictive value than other available variables	14	12	Age<br>Involuntary MHA status
4	Exclude any variable with IVs only achieving p>.100 across all outcome variables.	Limiting covariate inclusion to those with less predictive value than other available variables	12	9	Gender<br>HoNOS Q1<br>HoNOS Q5

Type	Predictor	Type	AUDIT	BPRS	HoNOS	LSP	MHI	SANS	SFS						
			RCI	RCI	RCI	RCI	RCI	RCI	RCI	***	**	*	^	~	Total
Time & Service	Admission date	Continuous			*			*				2			2
	CCU Length of stay (years)	Continuous			^		*					1	1		2
	Staffing model	Dichotomous	~	*	^				*			2	1	1	4
Consumer	Education	Scaled		*				*	~			2		1	3
	Primary Dx F20	Dichotomous	^	~	*							1	1	1	3
	Comorbid substance use	Dichotomous	~		^				~			1		2	3
	Comorbid PD	Dichotomous	*					~	~			1		2	3
Impairment at admission	HoNOS total	Continuous		~	***		^				1		1	1	3
	LSP-16 total	Continuous				***			^	1				1	2

~ p<.200-.100
^ p<.100-.050
* p<.050-.010
** p<.010-001
*** p<.001

The issue with this approach is that it results in 9 IVs for each outcome, some lacking clear predictive value across the outcome set.

Step 4a - Option B: **Focused covariates for each outcome**

	AUDIT	BPRS	HoNOS	LSP	MHI	SANS	SFS
	RCI	RCI	RCI	RCI	RCI	RCI	RCI
IV 1	Staffing model	Staffing model	Staffing model	Staffing model	Staffing model	Staffing model	Staffing model
IV 2	Gender	Education	Admission date	HoNOS Q1 (Aggression)	CCU Length of stay	Admission date	Education
IV 3	Primary Dx F20	Primary Dx F20	Gender	LSP-16 Total	Gender	Age	Comorbid substance use
IV 4	Comorbid substance use	Comorbid substance use	CCU Length of stay		HoNOS Total	Education	Comorbid PD
IV 5	Comorbid PD	HoNOS Total	Primary Dx F20		HoNOS Q1 (Aggression)	Comorbid PD	LSP-16 (Total)
IV 6	HoNOS Q5 (Physical)	HoNOS Q5 (Physical)	Comorbid substance use		HoNOS Q5 (Physical)		Involuntary MHA status
IV 7			HoNOS total				
IVs selected	6	6	7	2	5	4	6
Staffing added	No	No	No	Yes	Yes	Yes	No
Final IV count	6	6	7	3	6	5	6
Event rate (min)	27	44	70	68	48	25	36
Events / IV	4.5	7.3	10	22.7	8	5	6

- Staffing model is included in the modelling of all outcome given this is the principal variable from which we are interested in the impact of covariates.
- This approach focuses the modelling on the covariates most relevant to each outcome and reduces the number of the covariates to the point where the minimum 5 events per IV threshold is exceeded for 5 of the 6 outcomes, and the desirable target of at least 10 events per IV is exceeded for two of these five outcomes.
- However, this approach loses consistency across variables and a total of 13 separate IVs are considered across the outcomes.

<u>Step 4b.</u> Using F20.x rather than F20-29.x <u>and</u> excluding Comorbid personality disorder/issues as a covariate consideration

Final modelling solution for BPRS under the original approach resulted in very broad Exp(*B*) confidence intervals for 'Primary diagnosis F20-29.x' hence an alternative modelling approach where the diagnostic variable F20.x (yes/no) was considered. Note that 'Integrated Site A' was also removed from the covariate list based on the issues emerging during the previous modelling round when both this and the related variable (and principal IV focus) 'Integrated Staffing Model' had been included.

Additionally, comorbid personality disorder/issues were identified in only a very small proportion of the consumers (~7%) and this variable in the modelling for AUDIT RCI demonstrated an unacceptably broad confidence interval for Exp(*B*) (0.985-126.87). For these reasons an additional round of modelling was trialled excluding this as a covariate consideration

**Type**	**Predictor**	**Type**	**AUDIT**	**BPRS**	**HoNOS**	**LSP**	**MHI**	**SANS**	**SFS**						
			RCI	RCI	RCI	RCI	RCI	RCI	RCI	*******	******	*****	**^**	**~**	**Total**
Time & Service	Admission date	Continuous			*			*				2			2
	CCU Length of stay (years)	Continuous			^		*					1	1		2
	Staffing model	Dichotomous		*	^				*			2	1		3
Consumer	Age	Continuous													0
	Gender	Dichotomous			~		~							2	2
	Education	Scaled		*				*				2			
	Primary Dx F20	Dichotomous	~	~	*							1		2	3
	Comorbid substance use	Dichotomous	^		^				~				2	1	3
Impairment at admission	HoNOS total	Continuous		~	***		^			1			1	1	3
	HoNOS Q1 (aggression)	Scaled				~	^						1	1	2
	HoNOS Q5 (physical)	Scaled		~			^						1	1	2
	LSP-16 total	Continuous				***			^	1			1		2
	Involuntary MHA status	Dichotomous							^					1	1

~ p<.200-.100
^ p<.100-.050
* p<.050-.010
** p<.010-001
*** p<.001

- Apart from removal as Comorbid personality disorder/issue the altered IV set did not change covariates with p<.200 for BPRS, HoNOS, LSP, MHI
- For AUDIT RCI, Staffing model, Gender and HoNOS Item 5 no longer passed the p<.200 threshold
- For SANS RCI, Age no longer passed the p<.200
- For SFS RCI, Education level no longer pass the p<.200 threshold.

Step 5. Selecting the optimal modelling approach across the variables

Option A: Simultaneous entry of a standard set of IVs across all outcomes

TYPE	Predictor	AUDIT	BPRS	HoNOS	LSP	MHI	SANS	SFS
		RCI	RCI	RCI	RCI	RCI	RCI	RCI
Time & Service	Admission date			^			*	
	CCU LOS			^		*		~
	Integrated staffing model	+	^	^	+	+	+	*
Consumer	Education level		*				*	~
	Primary Dx F20-29.x		*	*				
	Comorbid substance use	^						
	Comorbid personality issue	^		~			~	^
Impairment	HoNOS admission total		*	***				
	LSP-16 admission total			~	***			
Summary	n p<.200	2	4	7	1	1	3	4
	n p<.100	2	4	5	1	1	2	1
	n p<.050	0	3	2	1	1	2	1

Key

+	p>.200 but included as primary IV focus
~	p>.200-.100
^	p>.100-.050
*	P>.050-.010
**	p>.010-001
***	p>.001

Option B: Focused selection of IVs (simultaneous entry) for each outcome based on p<.200 rule

Type	Predictor	AUDIT	BPRS	HoNOS#	LSP	MHI	SANS#	SFS
		RCI	RCI	RCI	RCI	RCI	RCI	RCI
Time & Service	Admission date			^			**	
	CCU Length of stay (years)			^		*		
	Staffing model	+	*	~	+	+	+	*
	Integrated site A		~					
Consumer	Age							
	Gender			^				
	Education		*				*	~
	Primary Dx F20-29.x		*	**	~			
	Comorbid substance use	^		~				~
	Comorbid PD	^					~	~
Impairment at admission	HoNOS total		*	***				
	HoNOS Q1 (aggression)				*	*		
	HoNOS Q5 (physical)							
	LSP-16 total				***			~
	Involuntary MHA status							~
Summary	n p<.200	0	1	2	1	0	3	6
	n p<.100	2	0	3	0	0	2	1
	n p<.050	0	4	2	2	2	2	1

Key

+	p>.200 but included as primary IV focus
~	p=.200-.100
^	p=.100-.050
*	p=.050-.010
**	p=.010-001
***	p=.001
#	No significant interactions identified between admission date and LOS, nor between Admission date and Staffing model, nor between gender and HoNOS total for HoNOS. Similarly, no significant interaction was identified between admission date and staffing model for SANS.

Option B was chosen as this approach identified a greater number of statistically significant predictors and meant that each model was actually tailored towards the outcome considered rather than the outcome set as a whole.

Step 5a. Selecting the optimal modelling approach across the variables using F20.x rather than F20-29.x as an IV

Note that only option B was considered based on the observations in Step 5 and the risk of overfitting with using a standard set of 9 IVs across all outcomes.

Type	Predictor	AUDIT	BPRS[a]	HoNOS[b]	LSP	MHI[c]	SANS[#]	SFS
		RCI	RCI	RCI	RCI	RCI	RCI	RCI
**Time & Service**	Admission date			^			**	
	CCU Length of stay (years)					*		
	Staffing model	+	^		+	+	+	*
**Consumer**	Age							
	Gender			^				
	Education		*				*	~
	Primary Dx F20.x	~	~	*				
	Comorbid substance use	~		*				~
	Comorbid PD	^					~	~
**Impairment at admission**	HoNOS total		*	***				
	HoNOS Q1 (aggression)				^	~		
	HoNOS Q5 (physical)							
	LSP-16 total				***			~
	Involuntary MHA status							~
**Interactions**	Length of stay * Staffing model			^				
**Summary**	n p<.200	2	1	-	-	1	1	
	n p<.100	1	1	2	1	-	-	
	n p<.050	-	2	2	-	1	1	
	n p<.010	-	-	-	-	-	1	
	n p<.001	-	-	1	1	-	-	

Key

- \+ p>.200 but included as primary IV focus
- ~ p=.200-.100
- ^ p=.100-.050
- * p=.050-.010
- ** p=.010-001
- *** p=.001
- a HoNOS Q5 was removed due to the significant correlation between this subscale and the total score, this altered the model outcome with HoNOS emerging as a p<.05 predictor and staffing model reducing from p<.05 to p<.10
- b No significant interactions identified between admission date and LOS, nor between Admission date and Staffing model. However, the interaction between Length of Stay * Staffing model demonstrated p<.10.
- c Given limited performance of HoNOS Total in the initial model at this round, this IV was dropped from the final model given the correlation between the HoNOS Total score and Items 1 and 5 of this measure that were included. This reduced the p-value (from .057 to .155) and CI for HoNOS item 1
- d No significant interaction was identified between admission date and staffing model for SANS

	AUDIT	BPRS	HoNOS	LSP	MHI	SANS	SFS
	RCI	RCI	RCI	RCI	RCI	RCI	RCI
IV 1	Staffing model	Staffing model	Staffing model	Staffing model	Staffing model	Staffing model	Staffing model
IV 2	Gender	Education	Admission date	HoNOS Q1 (Aggression)	CCU Length of stay	Admission date	Education
IV 3	Primary Dx F20	Primary Dx F20	CCU Length of stay	LSP-16 Total	Gender	Age	Comorbid substance use
IV 4	Comorbid substance use	Comorbid substance use	Gender		~~HoNOS Total~~	Education	Comorbid PD
IV 5	Comorbid PD	HoNOS Total	Primary Dx F20		HoNOS Q1 (Aggression)	Comorbid PD	LSP-16 (Total)
IV 6	HoNOS Q5 (Physical)	~~HoNOS Q5 (Physical)~~	Comorbid substance use		HoNOS Q5 (Physical)		Involuntary MHA status
IV 7			HoNOS total				
IVs selected	6	6	7	2	5	4	6
Staffing added	No	No	No	Yes	Yes	Yes	No
Final IV count	6	6	7	3	6	5	6
Event rate (min)	27	44	70	68	48	25	36
Events / IV	4.5	7.3	10	22.7	8	5	6

* On reviewing the model solutions a very broad Exp(B) CI was noted for Comorbid Personality Disorder for the AUDIT RCI outcome (0.985-126.87) given the very low frequency of comorbid personality disorder/issues on admission across the groups (~7%) and additional round of modelling was trialed excluding this as a covariate consideration.

Step 4b - Option A: Consistency of covariates across the outcomes

Given the large number of known potential confounders available in the data set relative to the sample size a target of 10 covariates was established (see Step 2). Decisions about which variables to include in the final analysis was guided by the following decision tree (with the aim of stopping when the target of ≤10 IVs was reached):

	Action	Rationale	Covariates		
			Start	End	Excluded
1	Include 'Staffing model' regardless of p-value	This is the IV of primary interest	13	13	N/A
2	Include any Level 1 (Time & Service) variable with p<.200 for an outcome variable	These variables are linked to the primary focus of the study and are relevant to understanding whether the impact of these organizational factors on the relationship between staffing model and outcomes (e.g. change in models over time, site-based variation between integrated sites).	13	13	N/A
3	Exclude any variable with only p<.200-.100 for a single IV across all outcomes	Limiting covariate inclusion to those with less predictive value than other available variables	13	12	Age
4	Exclude any variable with IVs only achieving p>.100 across all outcome variables.	Limiting covariate inclusion to those with less predictive value than other available variables	12	11	Gender

Type	Predictor	Type	AUDIT	BPRS	HoNOS	LSP	MHI	SANS	SFS						
			RCI	RCI	RCI	RCI	RCI	RCI	RCI	***	**	*	^	~	Total
Time & Service	Admission date	Continuous			*			*				2			2
	CCU Length of stay (years)	Continuous			^		*					1	1		2
	Staffing model	Dichotomous		*	^				*			2	1		3
Consumer	Education	Scaled		*				*				2			
	Primary Dx F20	Dichotomous	~	~	*							1		2	3
	Comorbid substance use	Dichotomous	^		^				~				2	1	3
Impairment at admission	HoNOS total	Continuous		~	***		^			1			1	1	3
	HoNOS Q1 (aggression)	Scaled				~	^						1	1	2
	HoNOS Q5 (physical)	Scaled		~			^						1	1	2
	LSP-16 total	Continuous				***			^	1			1		2
	Involuntary MHA status	Dichotomous							^					1	1

~ p<.200-.100
^ p<.100-.050
* p<.050-.010
** p<.010-001
*** p<.001

The issue with this approach is that it results in 11 IVs for each outcome, some lacking clear predictive value across the outcome set. High risk of overfitting.

Step 4b - Option B: Focused covariates for each outcome

	AUDIT	BPRS	HoNOS	LSP	MHI	SANS	SFS
	RCI	RCI	RCI	RCI	RCI	RCI	RCI
IV 1	Staffing model	Staffing model	Staffing model	Staffing model	Staffing model	Staffing model	Staffing model
IV 2	Primary Dx F20	Education	Admission date	HoNOS Q1 (Aggression)	CCU Length of stay	Admission date	Comorbid substance use
IV 3	Comorbid substance use	Primary Dx F20	CCU Length of stay	LSP-16 Total	Gender	Education	LSP-16 (Total)
IV 4		Comorbid substance use	Gender		HoNOS Total		Involuntary MHA status
IV 5		HoNOS Total	Primary Dx F20		HoNOS Q1 (Aggression)		
IV 6		HoNOS Q5 (Physical)	Comorbid substance use		HoNOS Q5 (Physical)		
IV 7			HoNOS total				
IVs selected	2	6	7	2	5	4	4
Staffing added	Yes	No	No	Yes	Yes	Yes	No
Final IV count	3	6	7	3	6	5	4
Event rate (min)	27	44	70	68	48	25	36
Events / IV	9	7.3	10	22.7	8	5	9

- Staffing model is included in the modelling of all outcome given this is the principal variable from which we are interested in the impact of covariates.
- This approach focuses the modelling on the covariates most relevant to each outcome and reduces the number of the covariates to the point where the minimum 5 events per IV threshold is exceeded for **all** outcomes, and the desirable target of at least 10 events per IV is exceeded for two of these five outcomes.
- However, this approach loses consistency across variables and a total of 12 separate IVs are considered across the outcomes.

Step 5b. Selecting the optimal modelling approach across the variables using F20.x rather than F20-29.x as an IV and excluding Comorbid personality disorder/issue as a covariate consideration

Note that only option B was considered based on the observations in Step 5 and the risk of overfitting with using a standard set of 9 IVs across all outcomes.

Type	Predictor	AUDIT	BPRS[a]	HoNOS[b]	LSP	MHI[c]	SANS[#]	SFS
		RCI	RCI	RCI	RCI	RCI	RCI	RCI
Time & Service	Admission date			*			*	
	CCU Length of stay (years)			^		*		
	Staffing model	+	^	~	+	+	+	*
Consumer	Gender			^				
	Education		*				*	
	Primary Dx F20.x	~	~	*				
	Comorbid substance use	~		^				~
Impairment at admission	HoNOS total		*	***				
	HoNOS Q1 (aggression)				^	~		
	HoNOS Q5 (physical)							
	LSP-16 total				***			~
	Involuntary MHA status							^
Summary	n p<.200	2	1	1	-	1	-	2
	n p<.100	-	1	3	1	-	-	1
	n p<.050	-	2	2	-	1	2	1
	n p<.010	-	-	-	-	-	-	
	n p<.001	-	-	1	1	-	-	

Key

- \+ p>.200 but included as primary IV focus
- ~ p=.200-.100
- ^ p=.100-.050
- * p=.050-.010
- ** p=.010-001
- *** p=.001
- [a] HoNOS Q5 was removed from the final model solution due to the HoNOS Total encompassing this item score. This improved the contribution of HoNOS (HoNOS Total and Item 5 achieved p=.200-.100 when both entered) and was superior to Item 5 when an alternative model was explored excluding the HoNOS total.
- [b] Interactions were explored between Admission date and CCU LOS and Staffing model. These not result in a superior solution to the initial model.
- [c] HoNOS total removed with the two individual HoNOS items retained (these performed favorably compared to modeling with HoNOS Total int their absence). Inclusion of an interaction between LOS*Staffing model was considered, but not included given very broad CI for Exp(*B*).

	AUDIT	BPRS	HoNOS	LSP	MHI	SANS	SFS
	RCI	RCI	RCI	RCI	RCI	RCI	RCI
IV 1	Staffing model	Staffing model	Staffing model	Staffing model	Staffing model	Staffing model	Staffing model
IV 2	Primary Dx F20	Education	Admission date	HoNOS Q1 (Aggression)	CCU Length of stay	Admission date	Comorbid substance use
IV 3	Comorbid substance use	Primary Dx F20	CCU Length of stay	LSP-16 Total	Gender	Education	LSP-16 (Total)
IV 4		Comorbid substance use	Gender		~~HoNOS Total~~		Involuntary MHA status
IV 5		HoNOS Total	Primary Dx F20		HoNOS Q1 (Aggression)		
IV 6		~~HoNOS Q5 (Physical)~~	Comorbid substance use		HoNOS Q5 (Physical)		
IV 7			HoNOS total				
IVs selected	2	~~6~~ 5	7	2	~~5~~ 4	4	4
Staffing added	Yes	No	No	Yes	Yes	Yes	No
Final IV count	3	~~6~~ 5	7	3	~~6~~ 5	5	4
Event rate (min)	27	44	70	68	48	25	36
Events / IV	9	8.8	10	22.7	9.6	5	9

References

1. Parker S, Arnautovska U, Siskind D, et al. Community-care unit model of residential mental health rehabilitation services in Queensland, Australia: predicting outcomes of consumers 1-year post discharge. *Epidemiology and Psychiatric Sciences* 2020; 29: e109. 2020/03/11. DOI: 10.1017/S2045796020000207.
2. Parker S, Dark F, Newman E, et al. Longitudinal comparative evaluation of the equivalence of an integrated peer-support and clinical staffing model for residential mental health rehabilitation: a mixed methods protocol incorporating multiple stakeholder perspectives. *BMC Psychiatry* 2016; 16: 179. 2016/06/04. DOI: 10.1186/s12888-016-0882-x.
3. Chatterjee S, Pillai A, Jain S, et al. Outcomes of people with psychotic disorders in a community-based rehabilitation programme in rural India. *The British journal of psychiatry : the journal of mental science* 2009; 195: 433-439. 2009/11/03. DOI: 10.1192/bjp.bp.108.057596.
4. De Girolamo G, Candini V, Buizza C, et al. Is psychiatric residential facility discharge possible and predictable? A multivariate analytical approach applied to a prospective study in Italy. *Social psychiatry and psychiatric epidemiology* 2014; 49: 157-167.
5. Gonda T, Deane FP and Murugesan G. Predicting clinically significant change in an inpatient program for people with severe mental illness. *The Australian and New Zealand journal of psychiatry* 2012; 46: 651-658. 2012/04/25. DOI: 10.1177/0004867412445527.
6. Grinshpoon A, Abramowitz MZ, Lerner Y, et al. Re-hospitalization of first-in-life admitted schizophrenic patients before and after rehabilitation legislation: a comparison of two national cohorts. *Social Psychiatry and Psychiatric Epidemiology* 2007; 42: 355-359. 2007/05/12. DOI: 10.1007/s00127-007-0167-2.
7. Killaspy H and Zis P. Predictors of outcomes for users of mental health rehabilitation services: a 5-year retrospective cohort study in inner London, UK. *Social Psychiatry and Psychiatric Epidemiology* 2013; 48: 1005-1012. 2012/09/05. DOI: 10.1007/s00127-012-0576-8.
8. Lim C, Barrio C, Hernandez M, et al. Recovery from schizophrenia in community-based psychosocial rehabilitation settings: rates and predictors. *Research on Social Work Practice* 2017; 27: 538-551.
9. Maxwell A, Tsoutsoulis K, Menon Tarur Padinjareveettil A, et al. Longitudinal analysis of statistical and clinically significant psychosocial change following mental health rehabilitation. *Disabil Rehabil* 2018: 1-13. 2018/07/07. DOI: 10.1080/09638288.2018.1482505.
10. Yoon J, Bruckner TA and Brown TT. The association between client characteristics and recovery in California's comprehensive community mental health programs. *American Journal of Public Health* 2013; 103: e89-e95.
11. Vittinghoff E and McCulloch CE. Relaxing the Rule of Ten Events per Variable in Logistic and Cox Regression. *American Journal of Epidemiology* 2007; 165: 710-718. DOI: 10.1093/aje/kwk052.
12. Field A. *Discovering statistics using IBM SPSS statistics*. sage, 2013.
13. Mickey RM and Greenland S. The impact of confounder selection criteria on effect estimation. *American journal of epidemiology* 1989; 129: 125-137.
