# Supplementary material for: Comparative Effectiveness of Integrated Peer Support and Clinical Staffing Models for Community-Based Residential Mental Health Rehabilitation: A Prospective Observational Study
Source: Community Ment Health J. 2022 Sep 3;59(3):459–70. doi: 10.1007/s10597-022-01023-8 (PMC9981709; doi:10.1007/s10597-022-01023-8)
Supplement: Supplementary file 2 — Supplementary file2 (PDF 250 KB) [file 10597_2022_1023_MOESM2_ESM.pdf]

Supplementary table 1. Comparison of total and subscale scores by CCU site (N = 145)

	Site 1 (clinical model, n=53)			Site 2 (integrated model, n=52)			Site 3 (integrated model, n=40)			Total			Kruskal-Wallis H test (df=2)	p value
	N	M	SD	N	M	SD	N	M	SD	N	M	SD		
**Functioning and disability**	-	-	-	-	-	-	-	-	-	-	-	-	-	-
HoNOS Total	53	8.98	6.13	51	9.75	4.71	40	12.80	6.64	144	10.31	5.99	9.444	.009
Behaviour	53	1.13	1.70	51	1.06	1.48	40	1.20	1.68	144	1.13	1.61	0.169	.919
Impairment	53	1.38	1.43	51	1.49	1.32	40	2.48	1.63	144	1.72	1.52	13.293	.001
Symptoms	53	2.74	2.16	51	3.65	2.33	40	4.55	2.50	144	3.56	2.41	12.303	.002
Social	53	3.74	2.99	51	3.55	2.77	40	4.58	3.16	144	3.90	2.97	2.740	.254
SFS Total	51	107.04	7.81	50	102.95	8.00	39	100.84	7.78	140	103.85	8.22	13.362	.001
Withdrawal / Social Engagement	51	100.10	8.27	50	98.11	11.80	39	96.67	7.51	140	98.43	9.54	3.950	.139
Interaction	51	117.86	18.90	50	109.12	18.46	40	108.48	17.38	141	112.10	18.71	8.374	.015
Prosocial	51	109.52	13.43	50	104.73	12.01	40	102.50	13.75	141	105.83	13.27	7.918	.019
Recreation	51	104.65	21.65	50	104.95	15.44	40	98.74	15.89	141	103.08	18.13	6.000	.050
Independence-Performance	51	109.25	10.35	50	101.54	13.19	40	99.06	12.72	141	103.63	12.78	16.618	.000
Independence-Competence	51	107.05	9.36	50	103.68	10.83	40	103.68	9.04	141	104.90	9.89	3.489	.175
Employment / Occupation	51	100.85	8.66	50	98.51	11.98	40	95.14	9.97	141	98.40	10.49	7.324	.026
Allen's Cognitive Level (ACL)	51	5.03	0.40	48	5.16	0.43	40	5.01	0.34	139	5.07	0.40	5.345	.069
LSP-16 Total[a]	53	10.70	5.74	51	12.53	6.17	40	13.98	6.14	144	12.26	6.11	6.309	.043
Withdrawal	53	3.42	2.37	51	4.24	2.56	40	4.83	2.63	144	4.10	2.56	7.091	.000
Self-care	53	3.38	2.14	51	4.53	2.41	40	5.48	2.25	144	4.37	2.41	17.767	.000
Compliance	53	2.13	1.54	51	2.10	1.65	40	1.88	1.64	144	2.05	1.60	0.840	.657
Anti-social	53	1.77	1.86	51	1.67	2.01	40	1.80	2.00	144	1.74	1.94	0.396	.821
**Symptoms**	-	-	-	-	-	-	-	-	-	-	-	-	-	-
BPRS Total	51	37.47	8.89	46	36.67	9.78	36	42.81	9.73	133	38.64	9.71	8.162	.017
Resistance	51	6.08	2.61	46	5.89	1.82	36	5.92	2.29	133	5.97	2.26	0.290	.865
Positive Symptoms	51	10.51	5.11	46	10.33	4.64	36	12.25	5.37	133	10.92	5.05	3.337	.189
Negative Symptoms	51	6.76	3.66	46	6.02	3.36	36	7.69	3.06	133	6.76	3.44	6.790	.034
Psychological discomfort	51	13.00	4.15	46	13.09	5.00	36	15.50	5.36	133	13.71	4.89	4.721	.094
SANS Total	51	43.53	18.88	49	49.53	16.69	36	50.61	18.24	136	47.57	18.09	3.992	.136
Affective flattening	52	14.37	8.90	50	15.00	8.24	37	15.03	8.25	139	14.77	8.44	0.122	.941
Alogia	51	3.29	4.12	50	5.02	4.28	36	5.58	4.13	137	4.53	4.27	10.231	.006
Avolition-Apathy	52	8.65	4.47	50	10.30	2.76	37	10.65	3.28	139	9.78	3.70	8.643	.013

SANS Anhedonia-Asociality	52	13.21	5.34	50	15.12	3.80	37	14.68	4.07	139	14.29	4.55	3.268	.195
Attention	52	3.44	3.32	49	4.43	3.48	37	4.84	2.81	138	4.17	3.28	4.564	.102
**Substance use**	-	-	-	-	-	-	-	-	-	-	-	-	-	-
AUDIT Total	48	4.90	7.86	50	10.38	10.11	35	6.80	6.96	133	7.46	8.84	12.468	.002
Consumption	50	2.30	2.93	50	4.40	3.31	35	2.91	2.62	135	3.24	3.12	6.640	.036
Dependence	50	0.80	2.19	50	1.84	3.26	35	0.74	1.52	135	1.17	2.55	9.673	.008
Alcohol-related problems	48	1.67	3.35	50	4.14	4.73	35	3.14	3.61	133	2.98	4.10	12.809	.002
**Psychological well-being**	-	-	-	-	-	-	-	-	-	-	-	-	-	-
Psychological MHI	52	146.44	32.28	52	138.48	39.64	40	146.43	34.55	144	143.56	35.67	1.601	.449
Psychological distress	48	67.50	21.31	48	67.50	21.31	38	66.71	24.49	135	68.39	23.76	0.849	.654
Psychological well-being	49	48.29	14.22	51	44.78	15.45	34	46.76	13.12	134	46.57	14.41	1.331	.514
Anxiety	52	27.19	9.36	52	28.88	10.61	40	27.25	9.85	144	27.82	9.93	0.513	.774
Depression	52	10.96	3.99	52	12.21	5.08	40	11.00	4.68	144	11.42	4.60	2.113	.348
Loss of beh / emot control	52	22.42	8.01	52	24.29	9.46	40	22.70	9.19	144	23.17	8.86	0.969	.616
General positive affect	52	34.27	9.84	52	32.08	10.85	40	34.85	8.93	144	33.64	9.98	2.513	.285
Emotional ties	52	7.02	2.80	52	6.46	3.03	40	6.40	2.50	144	6.65	2.80	1.326	.515
Life satisfaction	50	3.32	1.28	51	3.20	1.33	36	3.67	1.15	137	3.36	1.27	3.550	.169
**Recovery**	-	-	-	-	-	-	-	-	- -	-	-	-	-	-
STORI-30 mean category[b]	45	2.96	1.476	47	3.53	1.427	40	3.15	1.388	132	3.22	1.443	3.435	.180

[a] The results vary somewhat from the analysis reported in Parker et al. (2019) due to different coding of ratings 7 "Unable to rate" (n=3). In Parker et al. (2019), these three cases were excluded from the analysis, however, for the current analysis, a rating of 7 was considered to indicate absence or no difficulty in the specific area of functioning and was, therefore, re-coded into 0. As such, the current analysis of the LSP-16 is conducted on a sample of 53 consumers, while Parker et al. (2019) analysis was made on a sample of 50 consumers.

[b] Categories of recovery correspond to the following values: 1 - Moratorium; 2 - Awareness; 3 - Preparedness; 4 - Rebuilding; and 5 - Growth.

References

Burgess P, Pirkis J and Coombs T. (2009) Modelling candidate effectiveness indicators for mental health services. *Australian & New Zealand Journal of Psychiatry* 43: 531-538.

Parker S, Siskind D, Hermens DF, et al. (2019) A comprehensive cohort description and statistical grouping of community-based residential rehabilitation service users in Australia. *Frontiers in Psychiatry* 10: 798-813.
